# Chemical Profile and Biological Properties of Methanolic and Ethanolic Extracts from the Aerial Parts of *Inula britannica* L. Growing in Central Asia

**DOI:** 10.3390/molecules29235749

**Published:** 2024-12-05

**Authors:** Aktolkyn K. Ibadullayeva, Martyna Kasela, Kaldanay K. Kozhanova, Gulnara M. Kadyrbayeva, Jarosław Widelski, Krzysztof Wojtanowski, Aleksandra Józefczyk, Katarzyna Suśniak, Piotr Okińczyc, Meruyert I. Tleubayeva, Aigerim A. Karaubayeva, Moldir A. Zhandabayeva, Aigerim Z. Mukhamedsadykova, Anna Malm

**Affiliations:** 1 Department of Engineering Disciplines of Good Practices, School of Pharmacy, Kazakh National Medical University, 88 Tole Bi Street, Almaty 050012, Kazakhstan; ibadullaeva.ak@kaznmu.kz (A.K.I.); kozhanova.k@kaznmu.kz (K.K.K.); karaubayeva.a@kaznmu.kz (A.A.K.); mukhamedsadykova.a@kaznmu.kz (A.Z.M.); 2 Department of Pharmaceutical Microbiology, Medical University of Lublin, 1 Chodzki Street, 20-093 Lublin, Poland; katarzyna.susniak@umlub.pl (K.S.); anna.malm@umlub.pl (A.M.); 3 Department of Pharmacognosy with Medicinal Plants Garden, Lublin Medical University, 20-093 Lublin, Poland; jaroslaw.widelski@umlub.pl (J.W.); aleksandra.jozefczyk@umlub.pl (A.J.); 4 Independent Laboratory of Chemistry of Natural Products, Medical University of Lublin, 1 Chodzki Street, 20-093 Lublin, Poland; krzysztof.wojtanowski@umlub.pl; 5 Department of Pharmacognosy and Herbal Medicines, Wrocław Medical University, 211a Borowska Street, 50-556 Wrocław, Poland; piotr.okinczyc@umw.edu.pl; 6 Department of Organization and Management and Economics of Pharmacy and Clinical Pharmacy, Kazakh National Medical University, 88 Tole Bi Street, Almaty 050012, Kazakhstan; tleubayeva.m@kaznmu.kz; 7 Department of Pharmaceutical Technology, Kazakh National Medical University, 88 Tole Bi Street, Almaty 050012, Kazakhstan; zhandabaeva.m@kaznmu.kz

**Keywords:** *Inula britannica* L., chemical composition, HPLC/ESI-QTOF-MS, RP-HPLC/DAD, antioxidant activity, antimicrobial activity

## Abstract

The genus *Inula* has been used in folk medicine for centuries; however, the data concerning *Inula britannica* L. are scarce. This study aimed at investigating the chemical composition of methanolic and ethanolic extracts from the aerial parts of *I. britannica* collected in Kazakhstan and evaluating their antimicrobial and antioxidant properties, with special attention being paid to polyphenols. The total content of polyphenols and flavonoids in the extracts was determined colorimetrically, while their qualitative and quantitative analyses were conducted using HPLC/ESI-QTOF-MS and RP-HPLC/DAD. Their antioxidant potential was determined using the FRAP and DPPH methods, whereas their antimicrobial activity was determined by the microdilution method towards a panel of reference microorganisms, including pathogens of the human gastrointestinal tract. Chemical analysis demonstrated that the methanolic extract had a higher content of polyphenols (58.02 vs. 43.44 mg GAE/g) and flavonoids (21.69 vs. 13.91 mg QUE/g) than the ethanolic extract. In both extracts, 15 compounds were identified, with the highest contents being those of cynarine (13.96 and 11.68 mg/g) and chlorogenic acid (9.22 and 5.09 mg/g). The DPPH assay showed a higher antioxidant activity of the methanolic extract (19.78 ± 0.12 mg GAE/g) in comparison to that of the ethanolic extract (15.56 ± 0.24 mg GAE/g). Similarly, the FRAP method showed that the methanolic extract exerted a much higher antioxidant activity (5.07 ± 0.18 mmol Fe^2+^/g) than the ethanolic extract (0.39 ± 0.01 mmol Fe^2+^/g). In contrast, both extracts showed similar antimicrobial properties, with the highest activity being that against *Helicobacter pylori* ATCC 43504 (MIC = 0.125–0.25 mg/mL). This paper presents novel data on *I. britannica* L., implying its significance as a source of valuable active compounds and being a prerequisite for further biological studies.

## 1. Introduction

The genus *Inula* from the Asteraceae family, represented by over 100 species, is widely disseminated in Europe, Asia, and Africa [1]. Plants from the *Inula* genus are well known for their use in traditional medicine due to the rich biological activity provided by a high content of sesquiterpene lactones, diterpenes, triterpenes, and flavonoids, resulting in anticancer, antibacterial, antioxidative, hepaprotective, cytotoxic, and anti-inflammatory properties [1,2,3,4,5].

*Inula britannica* L., investigated in the present study, is a wild plant that has been used in folk medicine, especially in Chinese ethnomedicine, for centuries [6]. In Kazakhstan, 12 *Inula* species are growing all over the country, except for in the highlands, whereas *I. britannica* can be found in the west of Kazakhstan and in the Trans-Ili Alatau, as well as in the south of the Almaty region, where it is used as an anti-inflammatory, anthelmintic, wound-healing, restorative, antibacterial, hemostatic, and laxative medicinal plant [7,8]. The plant prefers the sandy loamy and clayey soil of eutrophic and disturbed grasslands. It is characterized by a 15–75 cm stem, yellow flowers, and often grows in the form of multiple plants gathered and connected through rhizomes [2].

*I. britannica* was shown to be a rich source of up to 102 different chemical compounds, isolated mainly from the aerial parts of the plant, including its flowers [2]. As documented previously, *Inula* species are rich in terpenoids, mostly sesquiterpene lactones, exhibiting a wide range of activities, including anticancer, antidiabetic, and analgesic activities. Although sesquiterpene lactones are characterized as cytotoxic, which limits the use of the plant, this particular feature gives them the potential to act against human parasites and plant pathogens [9]. For instance, *I. britannica* extracts exhibit acaricidal activity against *Tetranychus cinnabarinus*, a phytophagous mite endangering agricultural production [10]. Among the sesquiterpene lactones produced by the *Inula* plants are eudesmane, 1,10-secoeudesmane, germacrane, pseudoguaiane, eremophilane, and dimeric skeletons. Moreover, recent studies showed that *I. britannica* could be a source of new sesquiterpene lactones, exhibiting neuroprotective properties [11]. Other biologically active compounds isolated from *I. brittanica* are kaurane glycosides and flavonoids, such as quercetin, luteolin, and luteolin-7-O-glucoside, as well as steroids [12,13,14]. Despite numerous studies focusing on the *Inula* genus, the data concerning *I. britannica* are scarce or incomplete. Moreover, no data are available on the chemical composition and biological activity of plants collected in Kazakhstan.

Studies on plants used in ethnomedicine allow for not only the comprehension of the value and potential of native medicinal plants but also the assessment of the pharmacological profile and safety of their application in healthcare [15]. According to the current policy of the Republic of Kazakhstan and its development strategy, there is a need to increase the involvement of domestic medicinal plants in the national market, with special attention paid to plants used for centuries in folk medicine. This development priority is even more justified considering that the chemical composition of medicinal plants and their biological activity are often regionally dependent or even season-dependent [16,17].

For the above-mentioned reasons, this study aimed to determine and compare the chemical profile of methanolic and ethanolic extracts prepared from *I. britannica* collected in Kazakhstan and to characterize the spectrum of their biological activity, including their antioxidant and antimicrobial properties. Because polyphenols are generally considered as the most biologically active plant secondary metabolites, this study focused mostly on this particular group of compounds.

## 2. Results

### 2.1. Chemical Profile of Methanolic and Ethanolic Extracts from Inula britannica L.

As presented in Table 1, the content of polyphenols was expressed as gallic acid equivalents per g of dry extract (mg GAE/g), whereas the content of flavonoids was expressed as quercetin equivalents per g of dry extract (mg QUE/g). The methanolic *I. britannica* extract was characterized by a higher content of polyphenols and flavonoids. The concentration of polyphenols was 58.02 ± 1.44 mg GAE/g and 43.44 ± 1.14 mg GAE/g, whereas that of flavonoids was 21.69 ± 0.48 mg QUE/g and 13.91 ± 0.54 mg QUE/g for the methanolic and ethanolic extracts, respectively.

**Figure 1:**
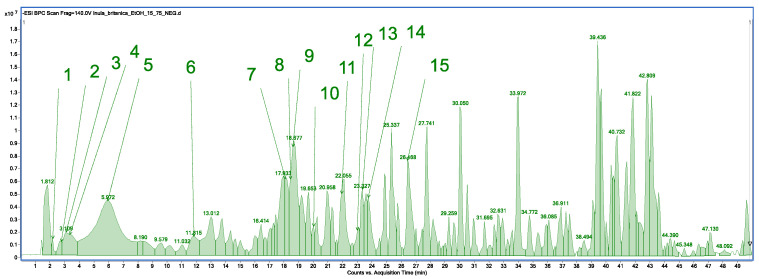
Base peak chromatogram of *Inula britannica* L. ethanolic extract by high-performance liquid chromatography–electrospray ionization–quadrupole time of flight–mass spectrometry (HPLC/ESI-QTOF-MS).

Qualitative analysis of the methanolic and ethanolic extracts from *I. britannica* showed the same chemical profile, consisting of 15 compounds. The list of compounds for the methanolic extract is presented in the Supplementary Materials (Table S1), while that for the ethanolic extract is presented below (Figure 1, Table 2). Additionally, all spectra for the identified compounds are available in the Supplementary Materials (Figure S1). In total, high-performance liquid chromatography–electrospray ionization–quadrupole time of flight–mass spectrometry (HPLC/ESI-QTOF-MS) identified 15 compounds from several classes of metabolites, mainly flavonoids, such as nepitrin.

Quantitative analysis of the *I. britannica* methanolic and ethanolic extracts with the use of the reversed-phase high-performance liquid chromatography–diode array detection (RP-HPLC/DAD) showed that in both the tested extracts, cynarine and chlorogenic acid were of the highest contents (Table 3, Figure S2). The concentration of cynarine was equal to 13.96 ± 0.1 mg/g and 11.68 ± 0.05 mg/g, whereas that of chlorogenic acid was 9.22 ± 0.03 mg/g and 5.09 ± 0.02 mg/g, respectively. Moreover, among the flavonoids, the nepitrin content was the highest, with 3.06 ± 0.04 mg/g in the methanolic extract and 1.9 ± 0.05 mg/g in the ethanolic extract. Statistical analysis showed that the content of neochlorogenic acid, chlorogenic acid, cynarin, nepitrin, and quercetin was significantly higher in the methanolic extract, whereas the content of caffeic acid glucoside, caffeic acid, and luteolin was significantly higher in the ethanolic extract.

### 2.2. Antioxidant Activity of Methanolic and Ethanolic Extracts from Inula britannica *L.*

The antioxidant activity of the methanolic and ethanolic extracts from *I. britannica* was investigated by the determination of the ferric reducing antioxidant power (FRAP) and using a 2,2-diphenyl-1-picrylhydrazyl assay (DPPH) (Table 4). Both methods showed that the methanolic extract had significantly higher antioxidant activity than the ethanolic one. The DPPH assay showed that the methanolic extract had significantly higher antioxidant activity (19.78 ± 0.12 mg GAE/g) in comparison to the ethanolic extract (15.56 ± 0.24 mg GAE/g). Similar differences in the antioxidant potential were observed based on the results obtained with the FRAP method, where the methanolic *I. britannica* extract had an activity of 5.07 ± 0.18 mmol Fe^2+^/g, 13 times higher than the ethanolic extract (0.39 ± 0.01 mmol Fe^2+^/g).

### 2.3. Antimicrobial Activity of Methanolic and Ethanolic Extracts from Inula britannica L.

The antimicrobial activity of the *I. britannica* methanolic and ethanolic extracts was tested against a wide panel of reference microorganisms, including Gram-positive bacteria, Gram-negative bacteria, and yeasts (Table 5). The panel included microbial species that cause a wide range of human infections, mostly species pathogenic to the human gastrointestinal tract.

The microbroth dilution method showed that both tested extracts demonstrated similar levels of antimicrobial activity—higher against the Gram-positive bacteria than the Gram-negative ones and yeasts. For all the Gram-positive species, the MIC (minimum inhibitory concentration) was equal to 2 mg/mL; however, the results differed in terms of the MBC (minimum bactericidal concentration), which varied from 2–4 mg/mL for the reference staphylococci (ATCC 12228, ATCC 29213, ATCC BAA-1707), *B. cereus* ATCC 10876, and *C. difficile* ATCC 43593 to 16 mg/mL for both *Enterococcus* strains (ATCC 29212 and ATCC 51299). No difference in the antibacterial activity was observed between the reference strains with acquired mechanisms of resistance, i.e., between methicillin-resistant and methicillin-sensitive *S. aureus* and between vancomycin-resistant and vancomycin-sensitive *E. faecalis*. The MBC-to-MIC ratio indicated that the extracts exhibited a bactericidal effect (MBC-to-MIC ratio ≤ 4), except for enterococci, where the dose required to kill bacterial cells was even eight times higher (16 mg/mL).

Both *I. britannica* extracts were characterized by a weak antimicrobial activity against most of the Gram-negative bacteria, including typical pathogens, such as *S. typhimurium* ATCC 14028, *L. monocytogenes* ATCC 19115, and *C. jejunii* ATCC 33560, except for *H. pylori* ATCC 43504, for which the MIC range was the lowest (0.125–0.25 mg/mL). Similar results were also obtained for three reference *Candida* species, where the MIC range was 8–16 mg/mL.

## 3. Discussion

Qualitative analysis of the methanolic and ethanolic extracts from *I. britannica* revealed the presence of the same chemical profile, consisting of fifteen compounds, including organic acids and their derivatives (malic acid, citric acid, neochlorogenic acid, caffeic acid glucoside, chlorogenic acid, coumarylquinic acid isomer), one dicaffeoylquinic acid (cynarine), and numerous flavonoids (patulitrin, nepitrin, hispuduloside, axillarin, quercetin, luteolin, nepetin, and kaempferol methyl ether). Studies have shown that multiple potentially bioactive compounds have been isolated from the genus *Inula*, e.g., from extracts prepared from the aerial parts of *I. hupehensis*, *I. falconeri*, or *I. hookeri*, whereas *I. britannica* chemical composition studies focus mostly on flowers [18]. There are only a few reports on the chemical composition of extracts prepared from all the aerial parts of *I. britannica* [19]. In general, the most important compound classes isolated from the *Inula* genus are eudesmanolides, guaianolides, pseudoguaianolides, germacranolides, xanthanolides, dimeric sesquiterpenes, and flavonoids [20].

Ivanova et al. investigated the chemical profile of an extract prepared from the flower heads of *I. britannica* of Bulgarian origin and revealed the presence of multiple sesquiterpene lactones (gaillardin, britannin, 11,13-dihydroinuchinenolide B, ivalin, and pulchellin C), triterpenoids (3-O-palmitates of 16β-hydroxylupeol, 16β-hydroxy-β-amyrin, and faradiol), and flavonoids (quercetin, luteolin, and luteolin-7-O-glucoside) [1]. Similarly, Bai et al. showed the presence of flavonoids in *I. britannica* extract (luteolin, diosmetin, chrysoeriol, kaempferol, quercetin, 6-hydroxyluteolin-6-methyl ether, spinacetin, and eupatin) [12]. This study and the previously mentioned studies prove that flavonoids constitute an important and, most of all, diversified group of secondary metabolites found in *I. brittanica*.

In the herein-investigated extracts, the predominant compounds were cynarine and chlorogenic acid. Cynarine and chlorogenic acid were detected previously in the *Inula* genus, in particular in *I. viscosa*, where the authors paid special attention to these two compounds as vasorelaxants, proving the antihypertensive effect of the plant extract [21]. Cynarin, a caffeoylquinic acid compound, often isolated from artichoke leaves (*Cynara scolymus* L.) [22,23], exhibits a wide range of biological properties, including antioxidant, anticholinergic, and metal-binding activities; however, no studies concerning its antimicrobial properties have been conducted to date [24]. Because it was the main compound isolated from the herein-investigated *I. britannica* extracts, further studies should also focus on providing novel information on the antibacterial and antifungal properties of cynarine. Compared with cynarine, chlorogenic acid is a well-studied antimicrobial compound. Chlorogenic acid, belonging to the class of polyphenols, is considered an active natural compound with multidimensional biological activity [25]. Among its antimicrobial properties documented before is the inhibition of the bacterial intracellular metabolism by downregulating the expression of genes involved in LPS (lipopolysaccharide) biosynthesis [26], the inhibition of biofilm formation [27], and the disruption of cell-to-cell bacterial communication (quorum sensing) [28]. It was also found to effectively act against a wide spectrum of bacteria and fungi, including human pathogens, such as *P. aeruginosa*, *Salmonella enteritidis*, *Klebsiella pneumoniae*, and *S. aureus*, as well as plant pathogens like *Fusarium* [29]. Moreover, it is also worth noting that one of the main flavonoids identified in this study in the extracts from *I. britannica*—nepitrin—was previously reported to exhibit anti-inflammatory properties [30].

Statistical analysis showed multiple differences between the methanolic and ethanolic *I. britannica* extracts in terms of the content of polyphenols and flavonoids, including particular compounds, as well as their antioxidant activity (*p* < 0.05). In our studies, the concentration of polyphenols ranged from 43.44 ± 1.14 mg GAE/g to 58.02 ± 1.44 mg GAE/g, whereas that of flavonoids ranged from 13.91 ± 0.54 mg QUE/g to 21.69 ± 0.48 mg QUE/g ± SD, and these were significantly higher in the methanolic extract. The significant differences in the chemical composition of the extracts can be explained by the more efficient extraction rate of particular compounds connected with the type of solvent used during the extraction. For instance, it has been shown that methanol is characterized by a higher extraction yield than ethanol, which is also visible in this study; thus, it is generally recommended as the best solvent to extract flavonoids and polyphenols from medicinal plants. Additionally, because plant extracts with a high content of polyphenols and flavonoids are found to possess antioxidant properties, the differences in the chemical composition between the methanolic and ethanolic *I. britannica* extracts also explain the significantly higher antioxidant activity of the extract obtained with the use of methanol [31]. Similar observations were made by other authors. Lee et al. studied the chemical profile of methanolic extract prepared from *I. britannica* flowers and demonstrated that the total polyphenol and flavonoid contents were 67.57 ± 0.08 mg GAE/g and 51.05 ± 0.42 mg QUE/g, respectively. Additionally, the authors showed that the main compounds were quercetin, naringenin, kaempferol, and hesperetin [32]. Also, Ceylan et al. showed slightly higher contents of polyphenols and flavonoids in *I. britannica* methanolic extract prepared from the plant’s aerial parts, where they were equal to 54.54 mg GAE/g and 30.98 mg QUE/g, respectively [33].

Different methods of measurement can quantify the antioxidant activity of plant extracts. It is generally recommended to use at least two different methods [34]. In this study, we used FRAP and DPPH assays, which showed that the methanolic *I. britannica* extract had significantly higher antioxidant activity than the ethanolic one. Currently, there is a lack of comprehensive data on the antioxidant activity of *I. britannica*. Ivanova et al. studied methanolic extracts of *I. britannica* collected in Bulgaria that were prepared from the flowers and the leaves of the plant with the use of a DPPH assay. The authors demonstrated that the antioxidant activity was higher in the methanolic extract prepared from the flowers, where it was equal to 37.6 µMT/g DM (Trolox equivalents per g of dry plant material), than that prepared from the leaves (13.7 µMT/g DM) [1]. Ceylan et al. conducted a comprehensive study documenting the substantial antioxidant activity of methanolic extracts obtained from the aerial parts of different *Inula* species, namely, *I. anatolica*, *I. britannica*, *I. inuloides*, *I. oculus-christi*, *I. peacockiana*, *I. sechmenii, I. thapsoides*, and *I. viscidula.* A DPPH assay showed a range of activity of 58.99–188.22 mg TE/g, while a FRAP assay showed a range of activity of 81.57–237.99 mg TE/g [33].

There are only a few studies investigating the microbiological activity of extracts or essential oils prepared from *I. britannica*. Moreover, they often focus on a narrow panel of microorganisms, mainly MRSA [32,35] and *Helicobacter pylori* [36], or they apply only screening techniques, i.e., disc/well diffusion methods, providing qualitative rather than quantitative data [37]. In our study, the methanolic and ethanolic *I. britannica* extracts exhibited higher antimicrobial activity against Gram-positive reference bacteria than Gram-negative ones and yeasts. Additionally, the MIC value was similar for antibiotic-sensitive and -resistant reference strains. The promising activity of various types of *I. britannica* extracts against MRSA was demonstrated in other studies, including extracts obtained by fermentation [32,35]. Na-Kyoung et al. investigated multiple clinical strains of MRSA, for which the MIC range of *I. britannica* extracts was 0.625–1.25 mg/mL, confirmed the bactericidal mode of action with a time–kill assay, and conducted SEM imaging, which revealed the influence of the tested extracts on MRSA’s cell morphology; the treated cells were shrunk and destroyed. Additionally, their study demonstrated that a methanolic extract prepared from the plant flowers significantly inhibited the expression of two genes involved in acquired β-lactam resistance—*mecA* and *mecRI* [32]. What is interesting is that the results of our studies suggest that for *E. faecalis* reference strains, the mode of action of *I. britannica* extracts might be bacteriostatic (MBC-to-MIC ratio equal 8) and not bactericidal, as was the case for the other tested Gram-positive bacteria. This difference could have multiple reasons, including different mechanisms of action of the tested extracts, which require further investigation.

The herein-observed activity against *Staphylococcus* spp., including MRSA as well as the results of the studies mentioned above, suggests that *I. britannica* may have the potential to be used in dermal applications in *Staphylococci*-associated infections of the skin as an alternative treatment or as an eradication agent in the case of *S. aureus* colonization. Several recent studies investigating *Inula* species have proven the potential of the extracts as candidates for the development of cosmetics, e.g., *I. salicina* [38], *I. helenium* [39], or *I. britannica* [40]. Moreover, *I. britannica* flower flavonoids were proven to exhibit antiaging effects in a mouse model induced by D-galactose [41]. However, to follow that direction, some essential studies should be conducted, including assessments of cytotoxicity and general safety towards human skin.

In our studies, the highest antibacterial activity was noted for *H. pylori* ATCC 43504 (MIC = 0.125–0.25 mg/mL). The antimicrobial activity of *I. britannica* against *H. pylori* was also documented by other authors, where the studies included both reference and clinical strains. Young Hwan et al. obtained low MIC values for methanolic and ethanolic extracts ranging from 0.075 to 0.1 mg/mL [36]. The authors demonstrated that the ethanolic extract had a stronger antimicrobial effect than the methanolic extract, which they explained by a higher content of quercetin, the solubility of which is higher in ethanol than in methanol. Moreover, the extracts at a concentration of 0.1 mg/mL reduced the activity of *H. pylori* urease by 20–30%, proving their potential as a part of an anti-virulence therapeutic strategy.

It is also worth mentioning that other *Inula* species have documented the ability to inhibit the formation of bacterial and fungal biofilm [42,43]. For example, research performed by Dimitrova et al. revealed that a methanolic extract from the aerial parts of *I. salicina* L., containing mainly chlorogenic acid and derivatives of dicaffeoylquinic acid, exhibited good activity against biofilm produced by *S. aureus*, *E. coli*, and *Pseudomonas aeruginosa*. The observed inhibitory effect was accompanied by morphological changes in the bacterial cells and their decreased viability [43].

In 2014, Seca et al. published a comprehensive and critical review on the chemical composition and bioactivity of the *Inula* genus, including *I. britannica*, confirming their significance as a rich reservoir of pharmacologically active compounds responsible for a wide spectrum of properties [20]. Recently, Malarz et al. underlined the importance of polyphenols as bioactive secondary metabolites of several *Inula* species related to their antioxidative, anti-inflammatory, and anticancer properties, together with pharmacological effects, such as reducing the glucose level and blood pressure, regulating adipogenesis, and counteracting depressive-like behavior [44]. Moreover, the biological and pharmacological effects of *I. viscosa*, such as antioxidant, anti-inflammatory, antifungal, antibacterial, antidiabetic, and antitumor activities, was reviewed by Ouari and Benzidane [45]. From the perspective of the pharmaceutical industry, it is worth noting that *I. japonica* Thunb. and *I. britannica* are included in the Chinese Pharmacopoeia (2020 edition) as the traditional Chinese medicine Flos Inulae [6].

## 4. Materials and Methods

### 4.1. Plant Material

The aerial parts of the *I. britannica* L. plant, consisting of stems and flowers, were collected at the flowering stage of the plant in the western part of Kazakhstan (50°44′54.8″ N 57°53′22.0″ E) in June 2019. The plant identification certificate (N 01-09/205-154) was issued by the specialists of the Institute of Botany and Phytointroduction of the Ministry of Ecology, Geology, and Natural Resources of the Republic of Kazakhstan. The raw material was dried at a temperature of 25 ± 5 °C in a well-ventilated room until the moisture content of the raw material dropped below 10%. Then, the material was crushed using an equipment-cutting mill (SM 300, Retsch, Haan, Germany) to obtain a particle size of 3–5 mm and stored in a sealed package at 15–25 °C and at a humidity of no more than 65%.

### 4.2. Preparation of Inula britannica L. Extracts

The pulverized plant material (aerial parts of *I. britannica*) was extracted with ethanol (96%, *v*/*v*) and methanol at a ratio of 1:10 (40 g of plant material per 400 mL of solvent). The extraction was performed in an ultrasonic bath (Sonorex, Bandelin, Berlin, Germany) at the following conditions: 20 °C (initial temperature) for 30 min and 756 W (90% of ultrasonic bath power). The obtained extracts were stored at room temperature for 12 h for stabilization and then filtered (Whatman No. 10 paper, Cytiva, Marlborough, MA, USA). Solvents were evaporated under reduced pressure, and extracts were frozen and lyophilized (Alpha 2-4 LD Plus lyophilizer, Christ, Osterode am Harz, Germany). The extraction yield was calculated as a gram of lyophilized extract per gram of dried plant material used for extraction. The extraction yield was 9.95% for the ethanolic extract and 12.35% for the methanolic extract. To avoid the negative influence of the solvents on the biological activity of the tested extracts, the solvents (methanol or ethanol) were evaporated (at 45 °C for 8 h; Concentrator plus, Eppendorf, Barkhausenweg, Germany), and the remaining content was used for further analysis.

### 4.3. Determination of Total Polyphenol Content and Total Flavonoid Content

The total polyphenol content was measured spectrophotometrically using the Folin–Ciocalteu test. Before the analysis, the dry extract solution and 50 µL of the ethanolic extract solution were mixed with 20 µL of Folin–Ciocalteu reagent. After 5 min, 200 µL of 100 g/L Na_2_CO_3_ solution was added. After 90 min of incubation at room temperature, in the dark, the absorbance was read against a blank (prepared similarly using pure solvent instead of sample) at 725 nm in disposable polystyrene 96-well plates (FL medical, Torreglia, Italy) using a microplate spectrophotometer (Multiskan™ GO Microplate Spectrophotometer; Thermo Fisher Scientific, Waltham, MA, USA). The results were calculated using fresh gallic acid standard solutions (10–200 µg/mL) and expressed as milligrams of gallic acid equivalent (GAE) per gram of dry extract.

The total flavonoid content was measured spectrophotometrically using a modified pharmacopeial method with aluminum chloride. An aliquot of 50 µL of dry extract solution was mixed with 50 µL of 2% ethanolic solution of AlCl_3_ (*w*/*v*), and after 60 min of incubation at room temperature, in the dark, the absorbance was measured at 420 nm using a microplate reader. The results were calculated using fresh quercetin standard solutions (20–400 µg/mL) and expressed as milligrams of quercetin equivalent (QUE) per gram of dry extract.

Every measurement was performed in triplicate. The standard deviation of the measurements was under 5%.

### 4.4. HPLC/ESI-QTOF-MS

The purified samples were analyzed qualitatively by an HPLC/ESI-QTOF-MS system in the negative ion mode with use of a 6530B Accurate-mass-QTOF-MS (Agilent Technologies, Inc., Santa Clara, CA, USA) mass spectrometer with an ESI-Jet Stream ion source. The Agilent 1260 chromatograph was equipped with a DAD detector, autosampler, binary gradient pump, and column oven (column Luna Omega Polar 100×, ø = 2.1 mm, particle size 3 µm; Phenomenex, Torrance, CA, USA). A gradient of solvents, i.e., water with 0.1% formic acid (solvent A) and acetonitrile with 0.1% formic acid (solvent B), were used as the mobile phases. The following gradient procedure was adopted: 0–45 min, 15–75% of B; 45–46 min, 75–95% B; 46–50 min 95% B; the post time was 10 min. The total time of the analysis was 60 min, with a stable flow rate at 0.200 mL/min. The injection volume for the extracts was 10 μL. ESI-QTOF-MS analysis was performed according to the following parameters of the ion source: dual spray jet stream ESI, positive and negative ion mode, gas (N_2_) flow rate: 12 L/min, nebulizer pressure: 35 psig, vaporizer temp.: 300 °C; m/z range: 50–1000 mass units, acquisition mode: Auto MS/MS, collision induced dissociation (CID): 10 and 30 eV with MS scan rate 1 spectrum per s, 2 spectra per cycle, skimmer: 65 V, fragmentor: 140 V, and octopole RF peak: 750 V. Identification of the compounds was performed with the use of previously published data and the MS DIAL software (version 4.70) [46,47].

### 4.5. RP-HPLC/DAD Analysis

The quantitative analysis of the *I. britannica* L. extracts was performed using RP-HPLC/DAD analysis. For the study, 0.03 g of extract from *I. britannica* L. was used, which was finally dissolved with small portions of a 2 mL methanol–water mixture (3:7 *v*/*v*) and transferred to an SPE microcolumn (C18 BAKERBOND SPE Octadecyl 500 mg; Avantor Performance Materials BV Deventer, The Netherlands) to purify the extracts from ballast compounds. The following analysis conditions were set: liquid chromatograph (Agilent Technologies, 1100, with a DAD detector and column thermostat at 250 °C); column: Zorbax Eclipse XDB C8 (150 × 4.6 mm × 5 μm); gradient elution with a flow of 1 cm^3^/min: water with 1% acetic acid (component A) and acetonitrile (component B), with an increasing concentration system of component B in A (from 0 min at 10% B to 50–55 min at 90% B). The identification of the investigated compounds from the HPLC analysis was based on the retention times, including those for the standard solutions, spectroscopically determining their spectra in UV (λ = 254, 280, and 325 nm). Based on the external standard method, the linearity of the quantitative procedure in this chromatographic method for the identified phenolic acids was evaluated. The data obtained from the chromatographic analysis (surface areas, retention times) were collected three times (*n* = 3), which allowed for the statistical processing of the data.

### 4.6. Antioxidant Activity

The total antioxidant activity (FRAP assay) and radical scavenging activity (DPPH test) were performed as described previously [48]. In the FRAP assay, the reagent was prepared by adding 10 mmol/L TPTZ reagent (2,4,6-tri(2-pyridyl)-s-triazine) to 20 mmol/L ferric chloride in acetate buffer (pH 3.6). Before the analysis, the tested extracts were diluted 20–200 times, and 20 μL of the extract solutions was mixed with 200 μL of the ferric complex. The experiment was conducted in 96-well plates (FL medical, Torreglia, Italy), and the absorbance was measured at 593 nm (Multiskan™ GO Microplate Spectrophotometer; Thermo Fisher Scientific, Waltham, MA, USA). The results were calculated using a calibration curve of ferrous sulfate (0.02–1.5 μmol/mL). In the DPPH assay, the extracts were diluted 20–200 times, and 20 µL of the diluted test extracts was mixed with 200 µL of 0.315 mM DPPH solution in methanol and incubated for 30 min at room temperature in the dark. The experiment was conducted in 96-well plates, and the absorbance was read at 517 nm using a microplate spectrophotometer. The results of the FRAP assay were presented as mmol of Fe^2+^ equivalents per gram of dry extracts, while those of the DPPH test were presented as gallic acid equivalents per gram of tested extracts. Every measurement was performed in triplicate. The standard deviation of the measurements was under 5%.

### 4.7. Antimicrobial Activity

The antimicrobial activity of the *I. britannica* extracts was determined using the microbroth dilution method according to the EUCAST recommendations [49]. The following parameters were determined: minimum inhibitory concentration (MIC), minimum bactericidal/fungicidal concentration (MBC/MFC), and the ratio of MBC/MIC or MFC/MIC, where a ratio ≤ 4 means that the extract exhibits bactericidal/fungicidal effect, while a ratio > 4 indicates a bacteriostatic/fungistatic effect. The activity was determined against the following non-fastidious microorganisms: bacteria: *Staphylococcus epidermidis* ATCC 12228, *S. aureus* ATCC 29213 (methicillin-susceptible *S. aureus*), *S. aureus* ATCC BAA-1707 (MRSA; methicillin-resistant *S. aureus*), *Enterococcus faecalis* ATCC 29212 (vancomycin-susceptible *Enterococcus*), *E. faecalis* ATCC 51299 (VRE; vancomycin-resistant *Enterococcus*), *Bacillus cereus* ATCC 10876, *Escherichia coli* ATCC 25922, *Pseudomonas aeruginosa* ATCC 27853, and *Salmonella typhimurium* ATCC 14028; yeasts: *Candida albicans* ATCC 10231, *C. glabrata* ATCC 90030, and *C. auris* CDC B11903. Bacteria were cultivated using Mueller–Hinton broth or agar (Biomaxima, Lublin, Poland), whereas for yeasts, the media were supplemented with 2% glucose. To avoid the negative influence of the solvents on the results of the antimicrobial analysis, the solvents (methanol or ethanol) were evaporated (at 45 °C for 8 h; Concentrator plus, Eppendorf, Hamburg, Germany), and the remaining content was resuspended in DMSO to obtain a stock concentration of 100 mg/mL that was stored at 4 °C. Then, directly before the analysis, the stock concentration was diluted to the desired initial concentration in an appropriate liquid medium. Briefly, the studied extracts were diluted two-fold in a 96-well microtiter plate (Nunc, Roskilde, Denmark) to obtain a range of concentration from 16 to 0.03125 mg/mL. Then, 100-fold-diluted 0.5 McFarland microbial suspension, prepared from overnight cultures, was added to each test well, giving a final concentration of microbial cells of 1.5 × 10^6^ CFU (colony-forming units)/mL for bacteria and 1.5 × 10^4^ CFU/mL for yeasts. Along with the test rows, multiple controls were included in the plate, namely, a positive control for each microbial strain, reassuring its appropriate growth, a negative control, confirming the sterility of the liquid media, and an extract control, consisting of serially diluted extract without bacterial cells. Plates were incubated for 24 h at 35 ± 2 °C, and after incubation, the absorbance in each well was spectrophotometrically measured at 600 nm (BioTek, Instruments, Winooski, VT, USA). Then, the MIC—the lowest extract concentration where no visible growth was observed in the well—was indicated by a visual assessment supported by comparing the absorbance between the test and extract control wells. To establish MBC/MFC, 5 µL of the well content was plated onto the solid medium. Then, the plates were incubated for 18–20 h at 35 ± 2 °C, and the MBC/MFC was set as the lowest extract concentration where no growth was observed after incubation.

The antimicrobial activity was also determined against fastidious bacteria pathogenic towards the gastrointestinal tract in humans, namely, *Clostridioides difficile* ATCC 43593 (pseudomembranous colitis), *Campylobacter jejunii* ATCC 29428 (campylobacteriosis), and *Listeria monocytogenes* ATCC 19115 (listeriosis). These strains were cultivated in Mueller–Hinton broth with 5% mechanically defibrinated horse blood and 20 mg/L β-NAD for 48 h at 35 ± 2 °C in an anaerobic atmosphere, 48 h at 41 ± 1 °C in a microaerophilic atmosphere, and 24 h at 35 ± 2 °C in aerobic conditions, respectively. The incubation atmosphere was modified by using chemical generators (GENbag anaer, GENbag microaer, bioMérieux, Craponne, France). Then, the MIC was read using a resazurin assay by adding 10 µL of 0.04% resazurin solution to each well and incubating in the above conditions for another 3 h. Because primarily blue resazurin turns pink due to the metabolic activity of microbial cells, the MIC was read as the lowest concentration where the color remained blue. The activity against *Helicobacter pylori* ATCC 43504 was determined as described before [50]. All microbiological assays described above were conducted in triplicate, and the results were presented as the mode.

### 4.8. Statistical Analysis

Statistical analysis of the results was conducted to compare the methanolic and ethanolic extracts from *I. britannica* in terms of the total content of polyphenols, flavonoids, the content of particular compounds, and their antioxidant properties measured with DPPH and FRAP assays. The normality of the distribution was checked using the Shapiro–Wilk W test; the distribution turned out to be close to normal only in slightly more than half of the cases; however, due to small sample size (*n* = 3), Student’s parametric *t*-test was used, and the statistical significance was set as *p* < 0.05.

## 5. Conclusions

The chemical composition and antimicrobial and antioxidant activity of methanolic and ethanolic extracts from *I. britannica* were reported in this paper. The chemical profile of both extracts consisted of 15 compounds, with the highest contents being those of cynarine and chlorogenic acid. The methanolic *I. britannica* extract showed a significantly higher total content of polyphenols and flavonoids and a significantly higher antioxidant activity than the ethanolic extract. Both extracts were similar in terms of their antimicrobial activity and acted most effectively against the reference *H. pylori* strain. Methanolic and ethanolic extracts obtained from *I. britannica* growing in its natural habitat in Kazakhstan are characterized by a unique chemical profile and constitute a valuable source of multiple bioactive compounds, especially flavonoids. Because of their antimicrobial activity, especially against *H. pylori*, the extracts should be further investigated to determine their cytotoxicity and other relevant biological properties.

## Figures and Tables

**Table 1:** Content of polyphenols and flavonoids in *Inula britannica* L. methanolic and ethanolic extracts.

*Inula britannica* L. Extracts	Polyphenols		Flavonoids	
	mg GAE/g ± SD	RSD	mg QUE/g ± SD	RSD
methanolic	58.02 ± 1.44 ^a^	2.48	21.69 ± 0.48 ^a^	2.19
ethanolic	43.44 ± 1.14 ^b^	2.62	13.91 ± 0.54 ^b^	3.85

SD—standard deviation; RSD—relative standard deviation; mg GAE/g—gallic acid equivalents per g of dry extract; mg QUE/g—quercetin equivalents per g of dry extract; different letters indicate statistically significant differences (*p* < 0.05).

**Table 2:** Results of ESI-QTOF-MS analysis of *Inula britannica* L. ethanolic extract.

No	Tentative Assignment	Rt [min]	Formula	Molecular Ion [*m*/*z*]	Error [ppm]	MS/MS Fragments [*m*/*z*]	PubChem CID
1	Malic acid	2.063	C_4_H_6_O_5_	133.0154	8.60	115.0033	525
2	Citric acid	2.130	C_6_H_8_O_7_	191.0557	−1.1	127.0406; 111.0081	311
3	Neochlorogenic acid	3.529	C_16_H_18_O_9_	353.0892	3.94	191.0503	5,280,633
4	Caffeic acid glucoside	3.796	C_15_H_18_O_9_	341.0908	8.75	179.0318; 161.0206; 135.0419	5,281,759
5	Chlorogenic acid	5.896	C_16_H_18_O_9_	353.0871	−1.99	191.0545	1,794,427
6	Coumarylquinic acid isomer	11.779	C_16_H_18_O_8_	337.1230	−1.19	191.0559	129,709,901
7	Patulitrin	17.412	C_22_H_22_O_13_	493.1034	9.38	331.0446; 316.0206; 287.0198; 181.0133	5,320,435
8	Cynarine	18.162	C_25_H_24_O_11_	515.1176	−3.68	353.0867; 191.0544; 179.0343	5,281,769
9	Nepitrin	18.212	C_22_H_22_O_12_	477.1031	−1.58	315.0558; 299.0182; 161.0233; 152.0102; 114.0547	120,742
10	Hispuduloside	19.845	C_22_H_22_O_11_	461.1091	0.89	298.0476; 283.0236; 255.0304; 161.0242; 137.0239	5,318,083
11	Axillarin	22.069	C_17_H_14_O_8_	345.0604	−3.44	330.0379; 315.0142; 287.0197; 271.0244; 243.0291	5,281,603
12	Quercetin	29.749	C_15_H_10_O_7_	301.0337	−5.55	178.9972; 151.0030; 121.0229; 107.0133	5,280,343
13	Luteolin	23.328	C_15_H_10_O_6_	285.0386	−6.51	199.0388; 175.0397; 151.0037; 133.0296; 107.0135	5,280,445
14	Nepetin	23.745	C_16_H_12_O_7_	315.0489	−6.73	300.0489; 243.0277; 228.0415; 216.0418; 165.9895; 136.9871	53,17,284
15	Kaempferol methyl ether	26.329	C_16_H_12_O_6_	299.0550	−3.7	284.0323; 256.0372; 227.0336; 151.0033	5,281,666

Rt—retention time.

**Table 3:** Results of the reversed-phase high-performance liquid chromatography–diode array detection (RP-HPLC/DAD) analysis of *Inula britannica* L. methanolic and ethanolic extracts.

No *	Tentative Assignment	λ [nm]	*Inula britannica* L. Extract			
			Methanolic		Ethanolic	
			mg/g ± SD	RSD	mg/g ± SD	RSD
3	Neochlorogenic acid	325	0.63 ± 0.01 ^a^	1.6	0.60 ± 0.01 ^b^	1.6
4	Caffeic acid glucoside	325	0.40 ± 0.01 ^a^	2.0	0.49 ± 0.00 ^b^	0.5
5	Chlorogenic acid	325	9.22 ± 0.03 ^a^	0.3	5.09 ± 0.02 ^b^	0.4
nd	Caffeic acid	325	1.02 ± 0.01 ^a^	1.3	1.31 ± 0.02 ^b^	1.2
8	Cynarine	325	13.96 ± 0.1 ^a^	0.7	11.68 ± 0.05 ^b^	0.4
9	Nepitrin	254	3.06 ± 0.04 ^a^	1.2	1.9 ± 0.05 ^b^	0.4
12	Quercetin	254	0.8 ± 0.01 ^a^	1.8	0.55 ± 0.02 ^b^	2.8
13	Luteolin	254	0.3 ± 0.00 ^a^	1.3	0.4 ± 0.01 ^b^	2.1

SD—standard deviation; RSD—relative standard deviation; * number corresponding to the results of qualitative analysis obtained with the ESI-QTOF-MS analysis; nd—not detected with ESI-QTOF-MS analysis; different letters indicate statistically significant differences (*n* = 3; *p* < 0.05).

**Table 4:** Antioxidant activity of methanolic and ethanolic extracts from *Inula britannica* L.

*Inula britannica* L. Extracts	FRAP		DPPH	
	mmol Fe^2+^/g ± SD	RSD	mg GAE/g ± SD	RSD
methanolic	5.07 ± 0.18 ^a^	3.46	19.78 ± 0.12 ^a^	0.60
ethanolic	0.39 ± 0.01 ^b^	3.29	15.56 ± 0.24 ^b^	1.56

SD—standard deviation; RSD—relative standard deviation; FRAP—ferric reducing antioxidant power; DPPH—2,2-diphenyl-1-picrylhydrazyl assay; mmol Fe^2+^/g—mmol Fe^2+^ equivalents per g of dry extract; mg GAE/g—gallic acid equivalents per g of dry extract; different letters indicate statistically significant differences (*p* < 0.05).

**Table 5:** Antimicrobial activity of methanolic and ethanolic extracts of *Inula britannica* L. (mg/mL).

Gram-Positive Bacteria	*Inula britannica* L. Extracts					
	Methanolic			Ethanolic		
	MIC	MBC	MBC/MIC	MIC	MBC	MBC/MIC
*Staphylococcus epidermidis* ATCC 12228	2	2	1	2	2	1
*Staphylococcus aureus* ATCC 29213	2	4	2	2	2	1
*Staphylococcus aureus* ATCC BAA-1707	2	4	2	2	4	2
*Enterococcus faecalis* ATCC 29212	2	16	8	2	16	8
*Enterococcus faecalis* ATCC 51299	2	16	8	2	16	8
*Bacillus cereus* ATCC 10876	2	2	1	2	2	1
*Clostridioides difficile* ATCC 43593	2	4	2	4	4	1
Gram-negative bacteria						
*Escherichia coli* ATCC 25922	16	16	1	16	16	1
*Salmonella typhimurium* ATCC 14028	16	16	1	16	16	1
*Pseudomonas aeruginosa* ATCC 27853	16	16	1	16	16	1
*Listeria monocytogenes* ATCC 19115	16	>16	>1	16	16	1
*Campylobacter jejunii* ATCC 33560	8	8	1	8	8	1
*Helicobacter pylori* ATCC 43504	0.25	nd	nd	0.125	nd	nd
Yeasts	MIC	MFC	MFC/MIC	MIC	MFC	MFC/MIC
*Candida albicans* ATCC 10231	8	16	2	8	16	2
*Candida glabrata* ATCC 90030	16	16	1	16	16	1
*Candida auris* CDC B11903	16	16	1	8	16	2

MIC—minimum inhibitory concentration; MBC—minimum bactericidal concentration; MFC—minimum fungicidal concentration; nd—not determined.

## Data Availability

Data are available from the corresponding authors.

## Supplementary Materials

The following supporting information can be downloaded at: https://www.mdpi.com/article/10.3390/molecules29235749/s1, Table S1: Base peak chromatogram of *Inula britannica* L. ethanolic extract by high-performance liquid chromatography–electrospray ionization–quadrupole time of flight–mass spectrometry (HPLC/ESI-QTOF-MS) and the results of ESI-QTOF-MS analysis of *Inula britannica* L. ethanolic extract. Figure S1: Fragmentation data of the tentatively identified components of methanolic and ethanolic extracts obtained from *Inula britannica* L. Figure S2: RP-HPLC/DAD chromatograms of *Inula britannica* L. methanolic and ethanolic extracts.
