# Supplementary material for: Chemical Profile and Biological Properties of Methanolic and Ethanolic Extracts from the Aerial Parts of Inula britannica L. Growing in Central Asia
Source: Molecules. 2024 Dec 5;29(23):5749. doi: 10.3390/molecules29235749 (PMC11643507; doi:10.3390/molecules29235749)
Supplement: Supplementary file 1 [file molecules-29-05749-s001.zip › molecules-3315065-supplementary.pdf]

Figure S1. Fragmentation data of the tentatively identified components of methanolic and ethanolic extracts obtained from *Inula britannica* L. The marked peak corresponds to m/z value of molecular ion.

A. Ethanolic *Inula britannica* L. extract

1.

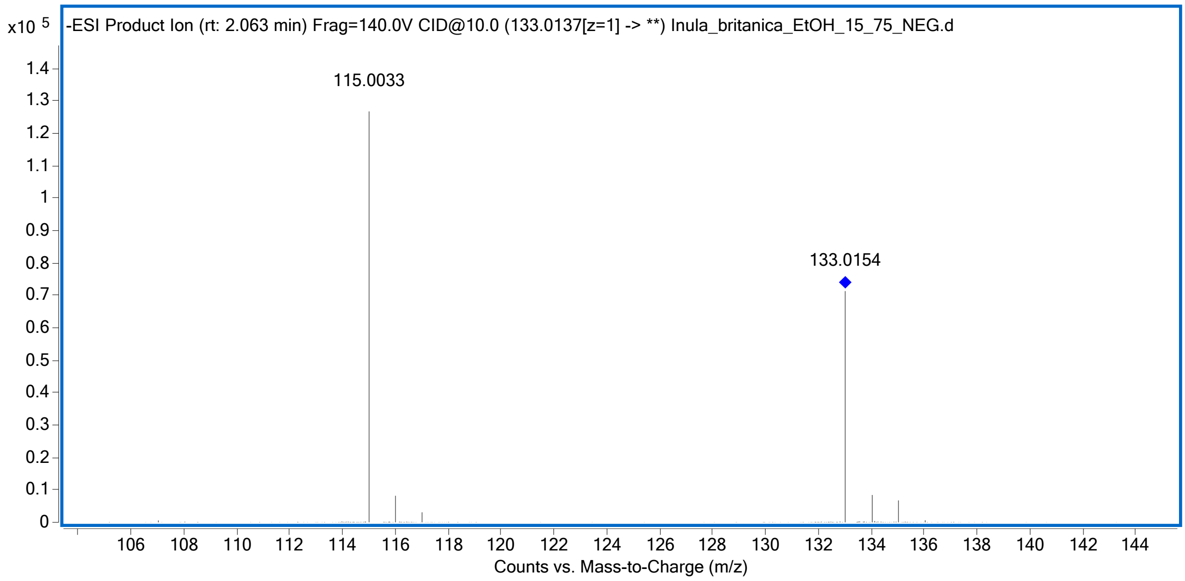

2.

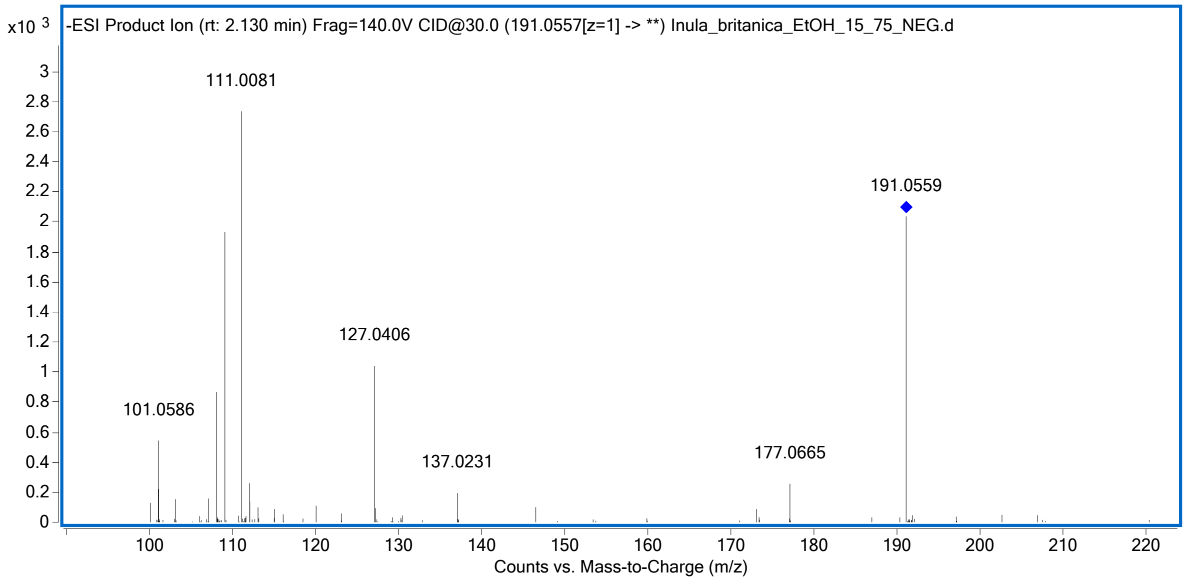

3.

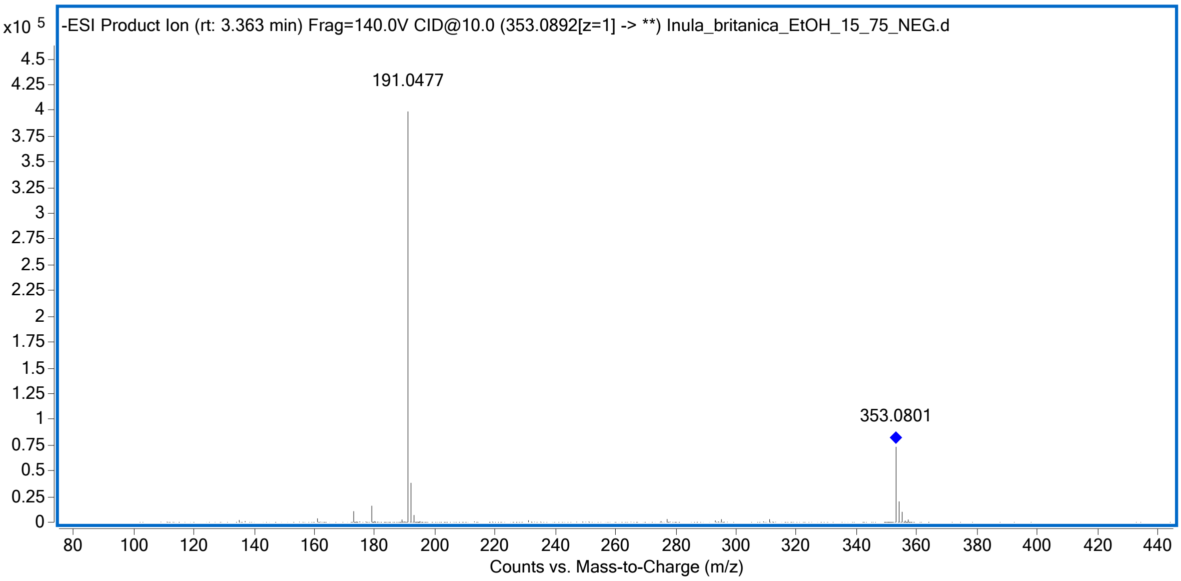
x10 5
-ESI Product Ion (rt: 3.363 min) Frag=140.0V CID@10.0 (353.0892[z=1] -> **) Inula_britanica_EtOH_15_75_NEG.d
191.0477
353.0801
Counts vs. Mass-to-Charge (m/z)

4.

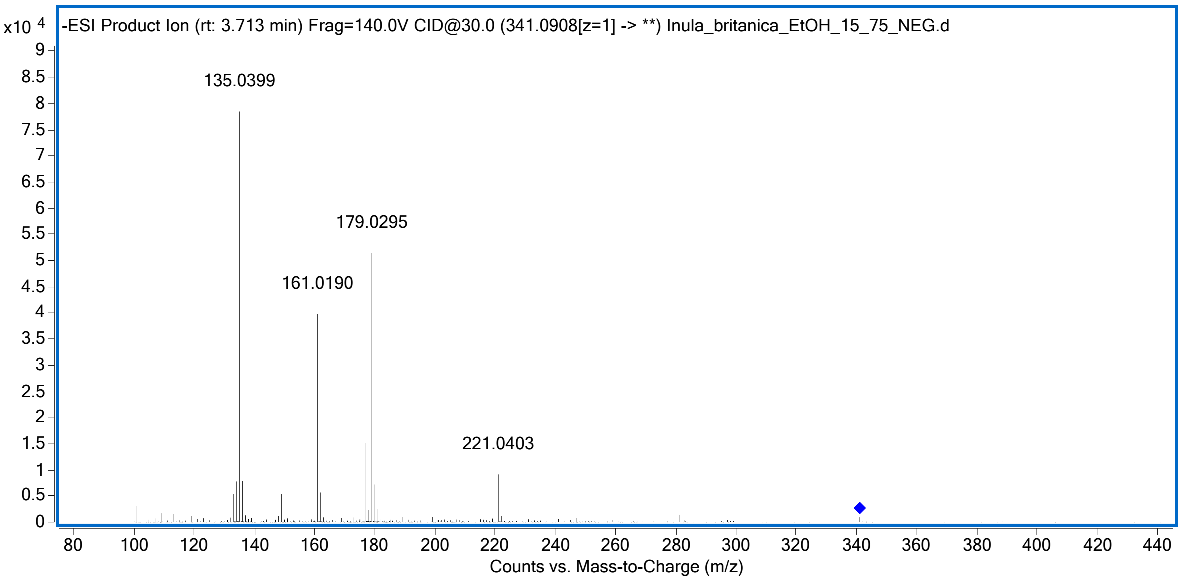
x10 4
-ESI Product Ion (rt: 3.713 min) Frag=140.0V CID@30.0 (341.0908[z=1] -> **) Inula_britanica_EtOH_15_75_NEG.d
135.0399
179.0295
161.0190
221.0403
Counts vs. Mass-to-Charge (m/z)

5.

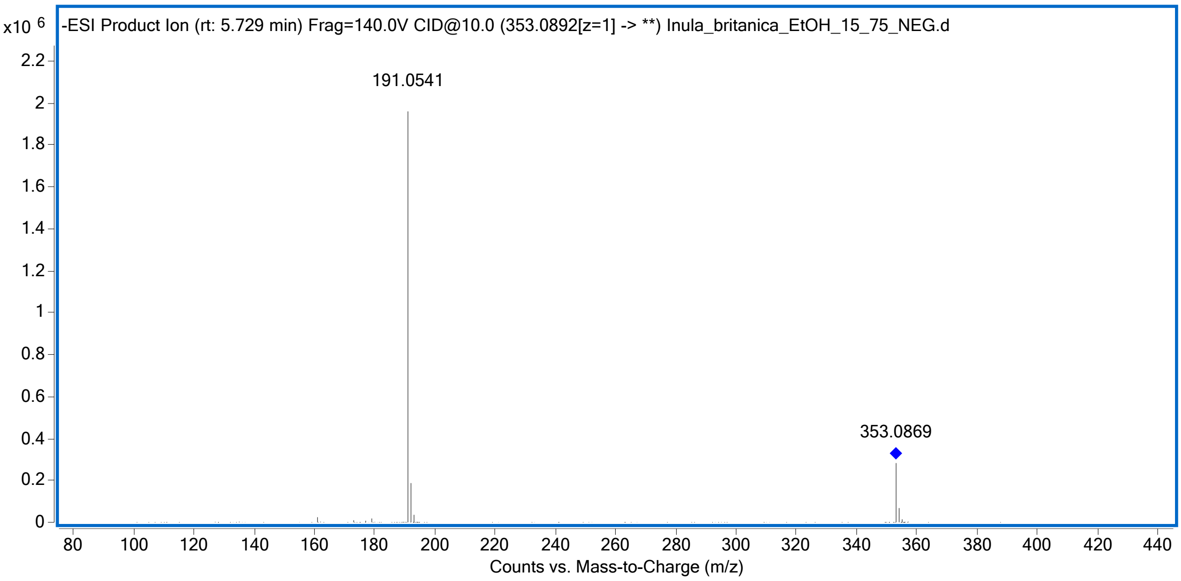
x10 6
-ESI Product Ion (rt: 5.729 min) Frag=140.0V CID@10.0 (353.0892[z=1] -> **) Inula_britanica_EtOH_15_75_NEG.d
191.0541
353.0869
Counts vs. Mass-to-Charge (m/z)

6.

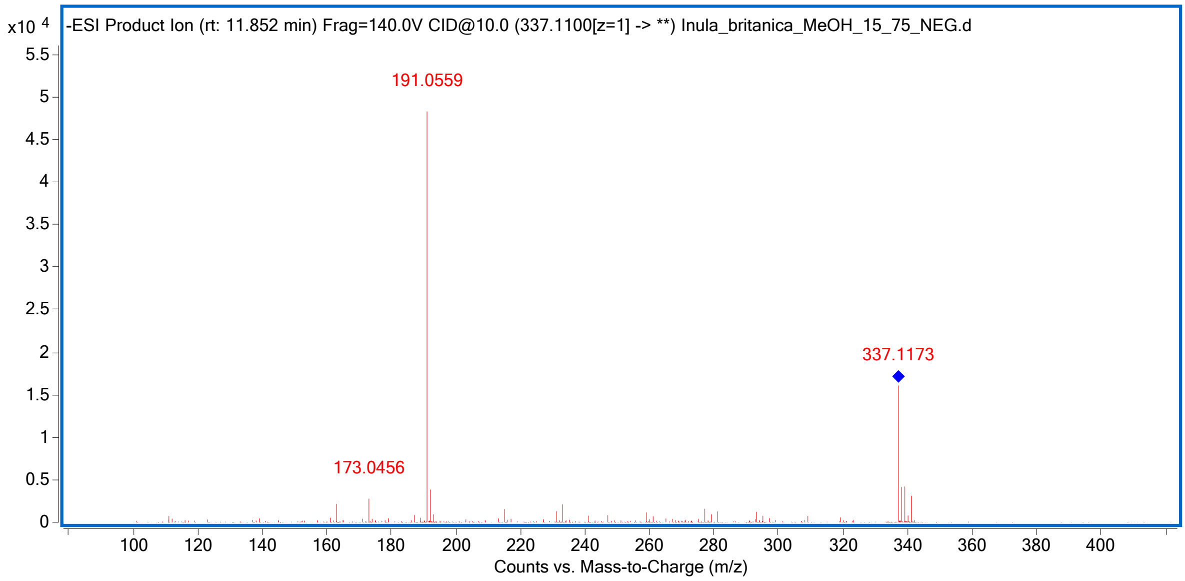
-ESI Product Ion (rt: 11.852 min) Frag=140.0V CID@10.0 (337.1100[z=1] -> **) Inula_britanica_MeOH_15_75_NEG.d
x10 4
191.0559
337.1173
173.0456
Counts vs. Mass-to-Charge (m/z)

7.

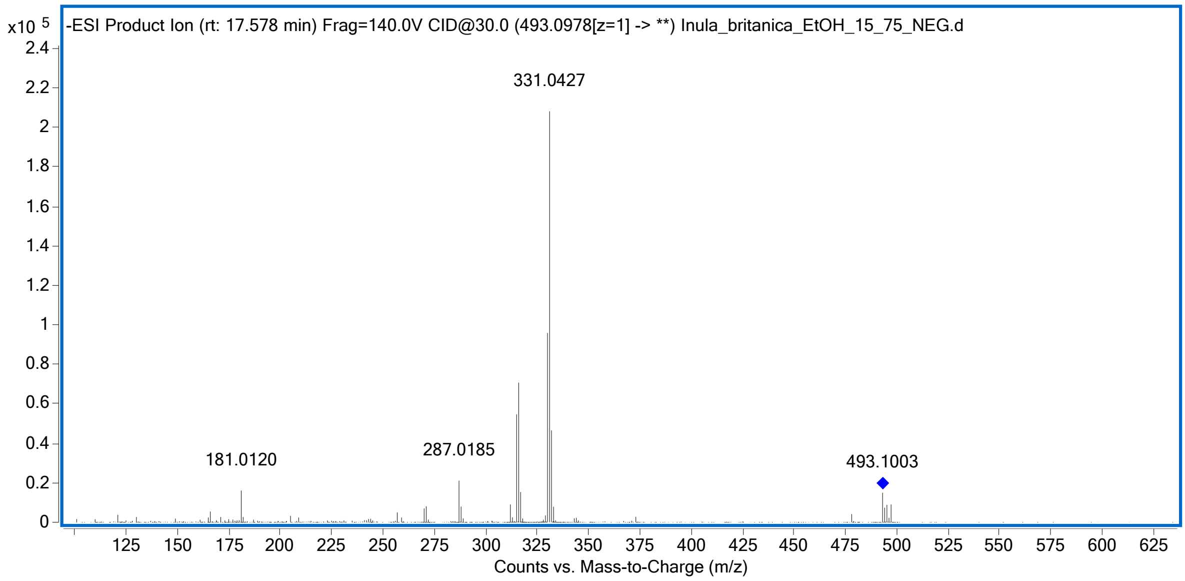
-ESI Product Ion (rt: 17.578 min) Frag=140.0V CID@30.0 (493.0978[z=1] -> **) Inula_britanica_EtOH_15_75_NEG.d
x10 5
331.0427
181.0120
287.0185
493.1003
Counts vs. Mass-to-Charge (m/z)

8.

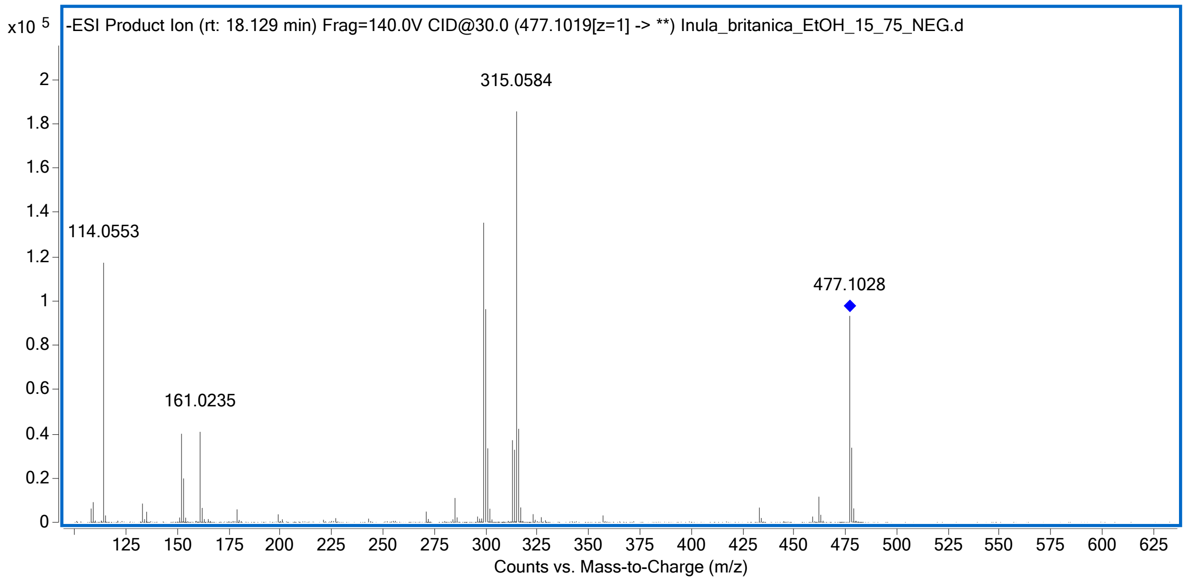
-ESI Product Ion (rt: 18.129 min) Frag=140.0V CID@30.0 (477.1019[z=1] -> **) Inula_britanica_EtOH_15_75_NEG.d
x10 5
315.0584
114.0553
477.1028
161.0235
Counts vs. Mass-to-Charge (m/z)

9.

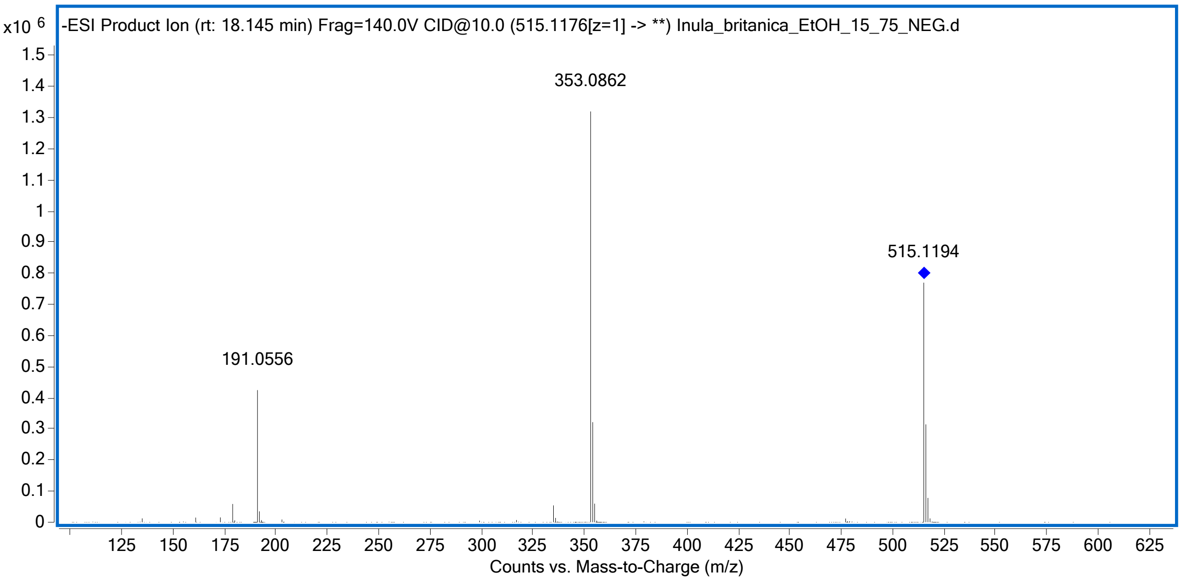
x10 6
-ESI Product Ion (rt: 18.145 min) Frag=140.0V CID@10.0 (515.1176[z=1] -> **) Inula_britanica_EtOH_15_75_NEG.d
353.0862
515.1194
191.0556
Counts vs. Mass-to-Charge (m/z)

10.

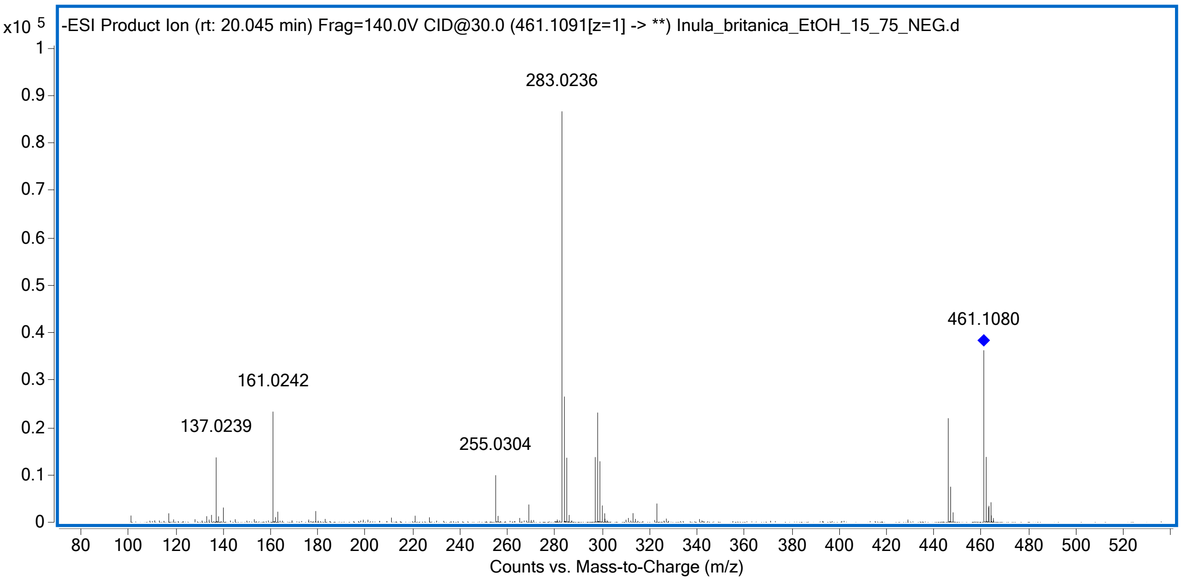
x10 5
-ESI Product Ion (rt: 20.045 min) Frag=140.0V CID@30.0 (461.1091[z=1] -> **) Inula_britanica_EtOH_15_75_NEG.d
283.0236
461.1080
161.0242
137.0239
255.0304
Counts vs. Mass-to-Charge (m/z)

11.

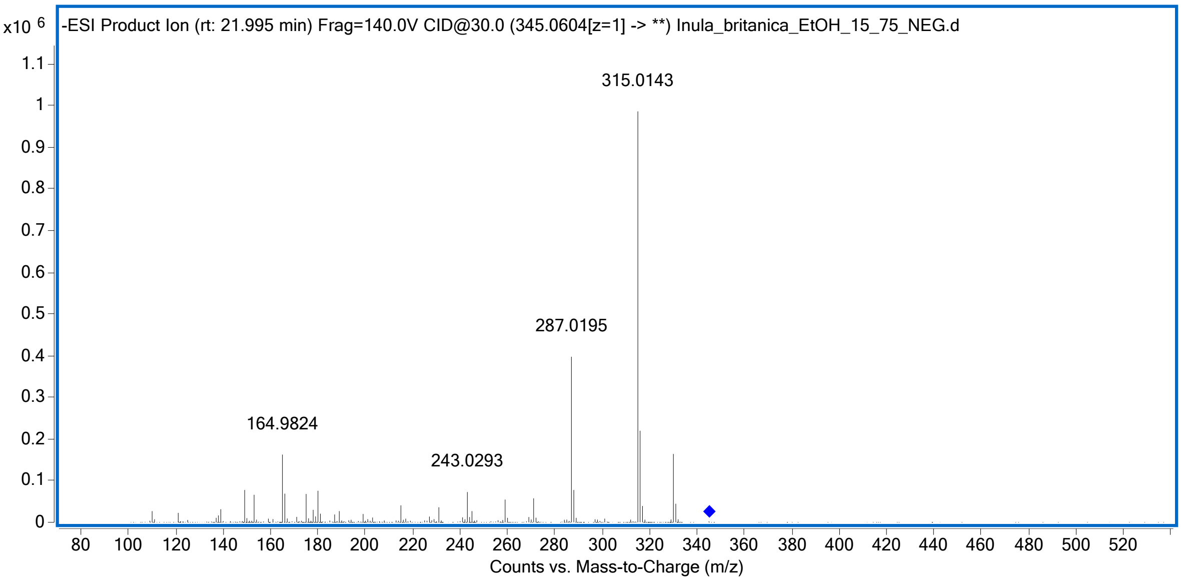
x10 6
-ESI Product Ion (rt: 21.995 min) Frag=140.0V CID@30.0 (345.0604[z=1] -> **) Inula_britanica_EtOH_15_75_NEG.d
315.0143
287.0195
164.9824
243.0293
Counts vs. Mass-to-Charge (m/z)

12.

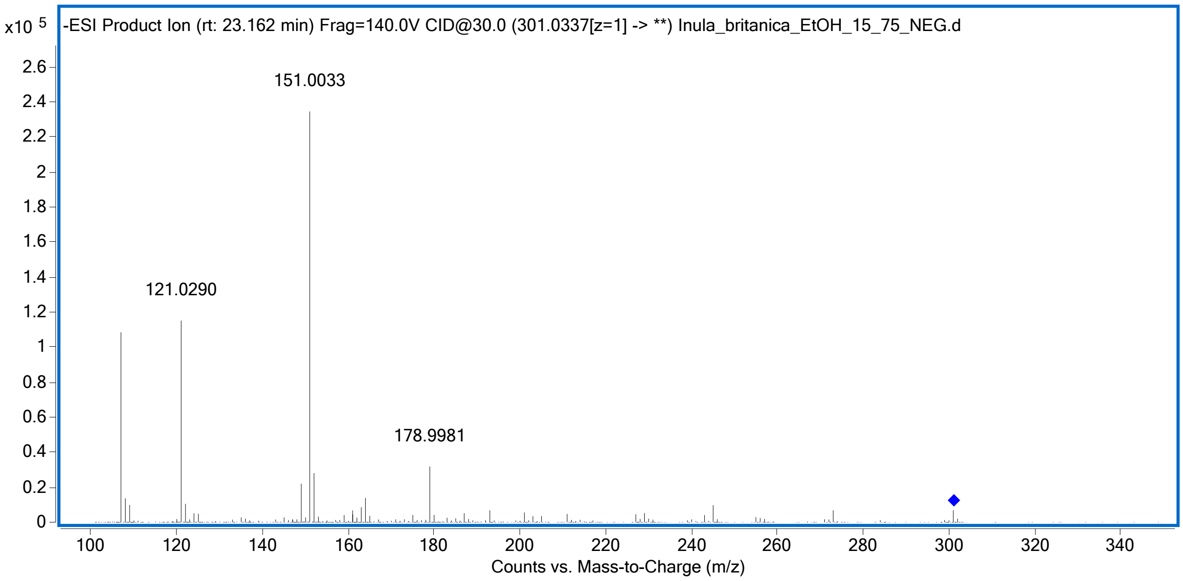
x10 5
-ESI Product Ion (rt: 23.162 min) Frag=140.0V CID@30.0 (301.0337[z=1] -> **) Inula_britanica_EtOH_15_75_NEG.d
151.0033
121.0290
178.9981
Counts vs. Mass-to-Charge (m/z)

13.

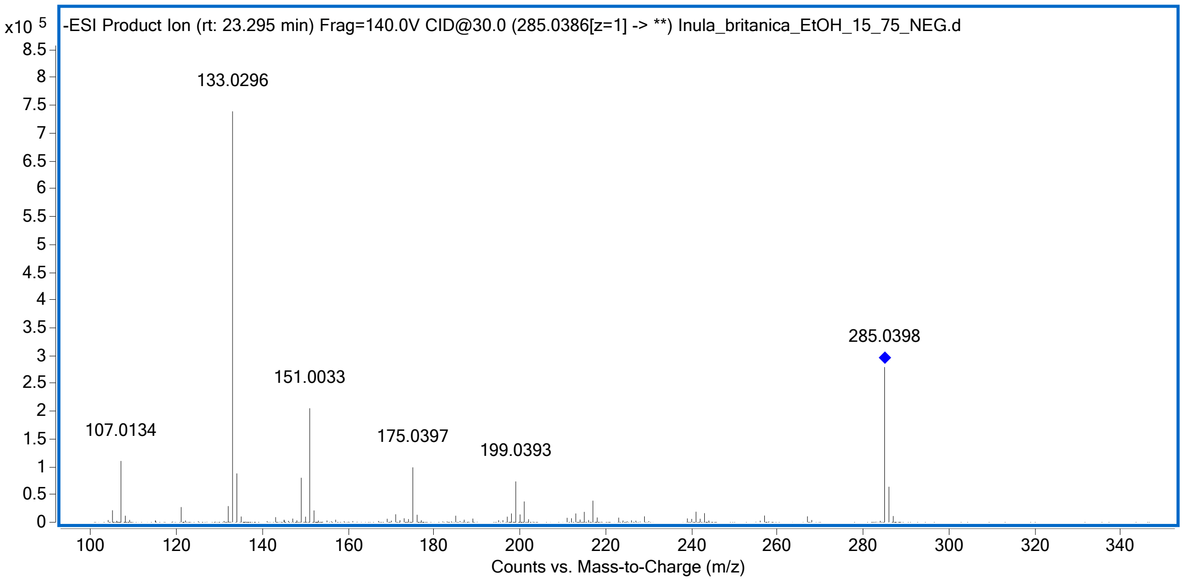
x10 5
-ESI Product Ion (rt: 23.295 min) Frag=140.0V CID@30.0 (285.0386[z=1] -> **) Inula_britanica_EtOH_15_75_NEG.d
133.0296
285.0398
151.0033
107.0134
175.0397
199.0393
Counts vs. Mass-to-Charge (m/z)

14.

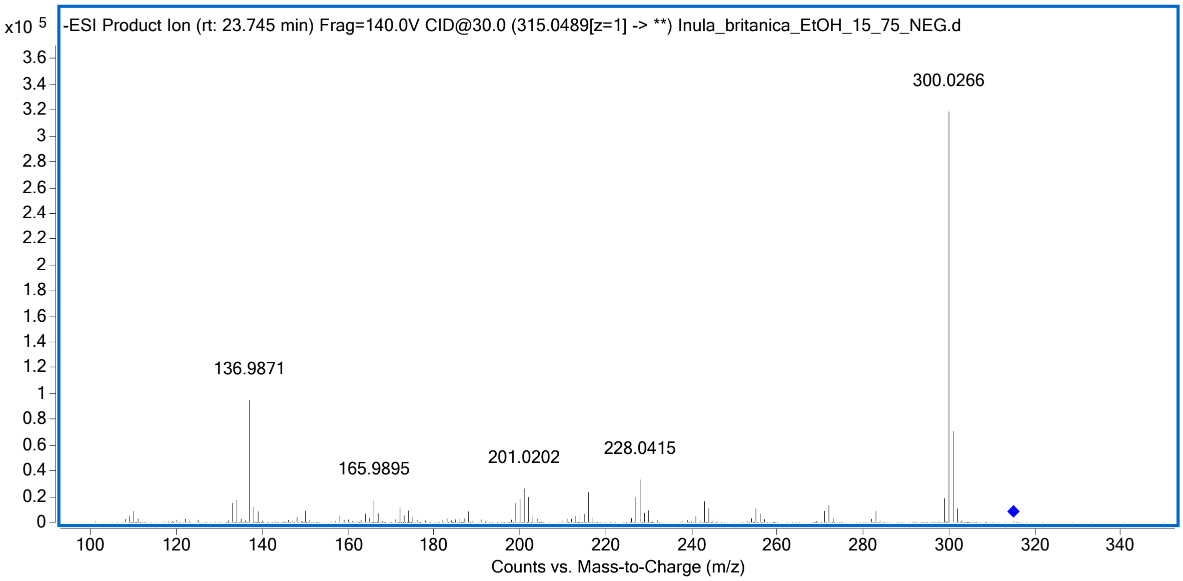
x10 5
-ESI Product Ion (rt: 23.745 min) Frag=140.0V CID@30.0 (315.0489[z=1] -> **) Inula_britanica_EtOH_15_75_NEG.d
300.0266
136.9871
165.9895
201.0202
228.0415
Counts vs. Mass-to-Charge (m/z)

15.

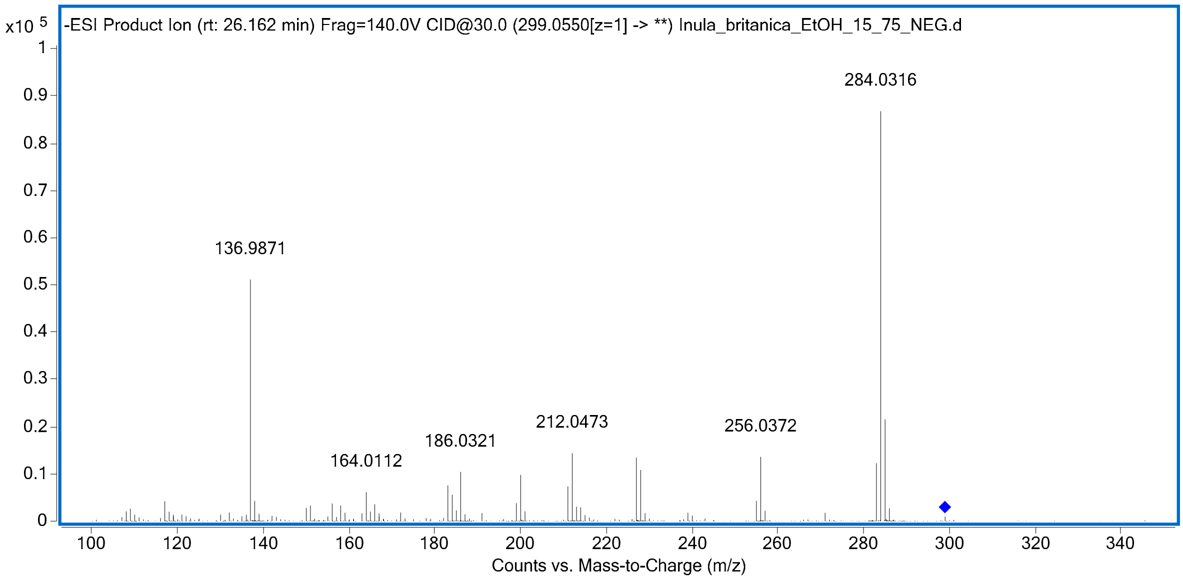

B. Methanolic *Inula britannica* L. extract

1.

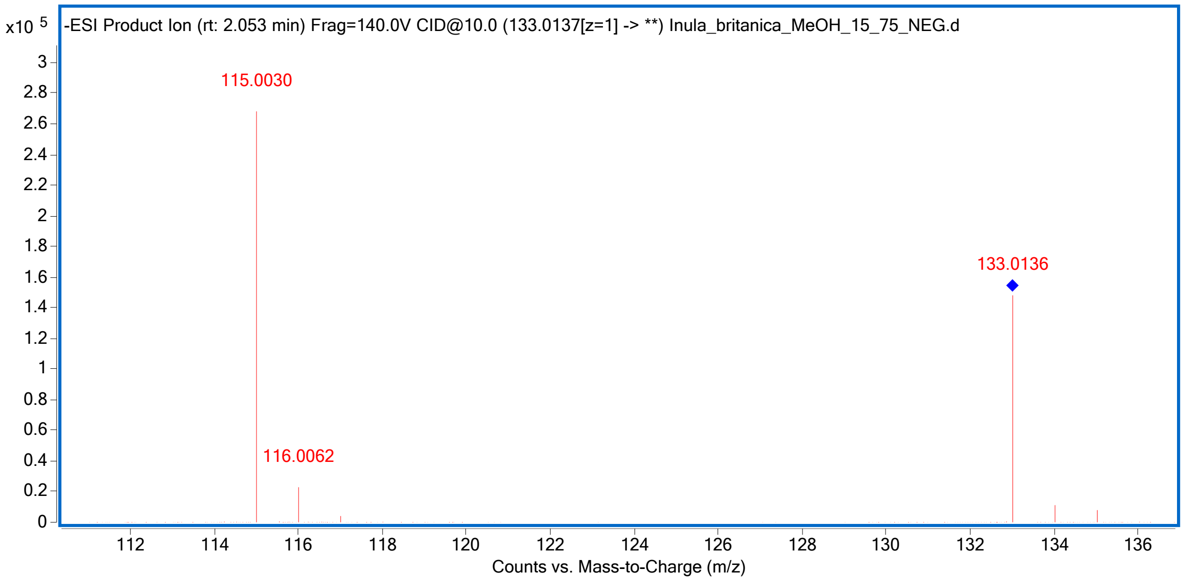

2.

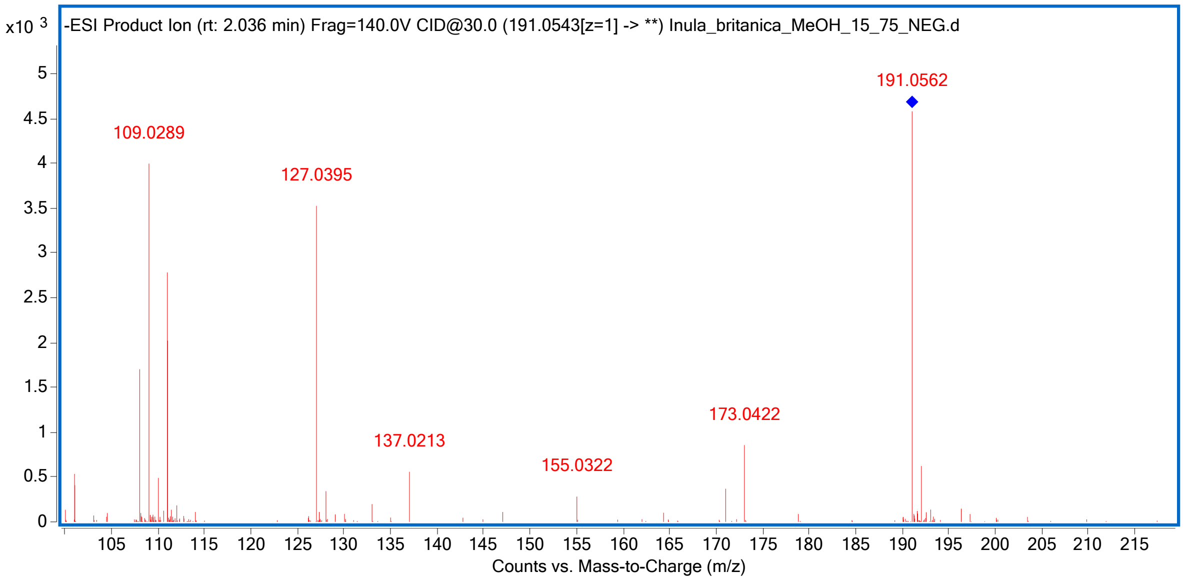

3.

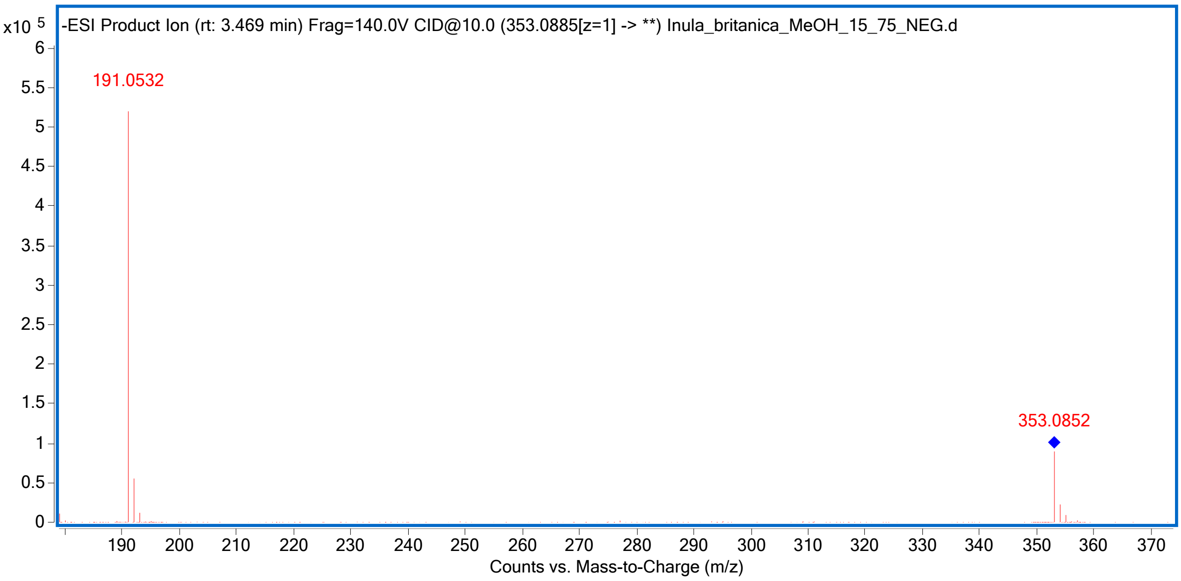
x10 5
-ESI Product Ion (rt: 3.469 min) Frag=140.0V CID@10.0 (353.0885[z=1] -> **) Inula_britanica_MeOH_15_75_NEG.d
191.0532
353.0852
Counts vs. Mass-to-Charge (m/z)

4.

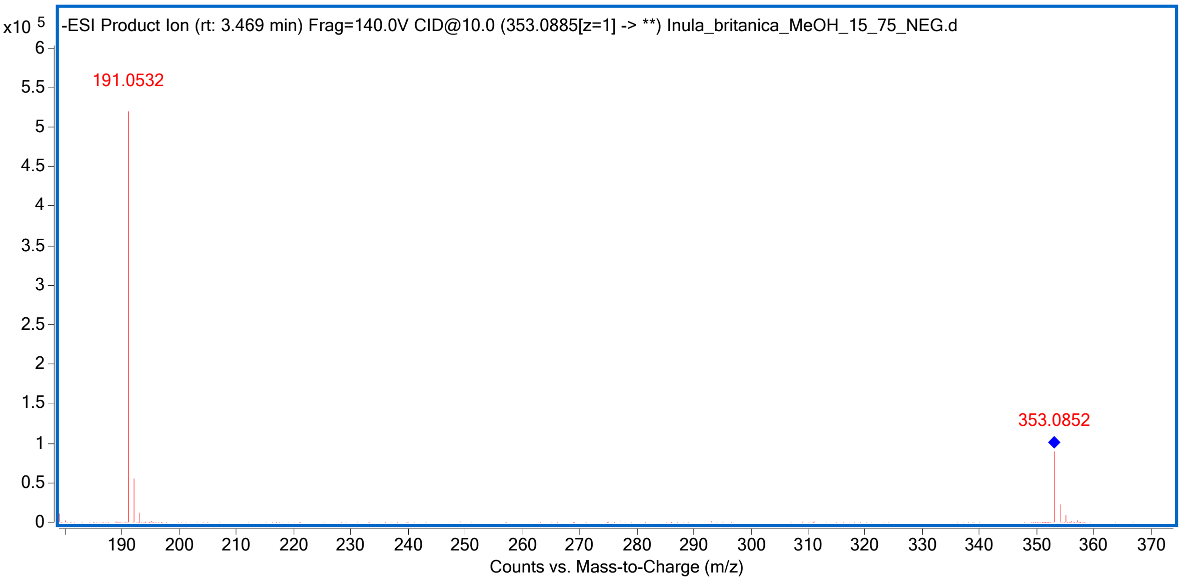
x10 5
-ESI Product Ion (rt: 3.469 min) Frag=140.0V CID@10.0 (353.0885[z=1] -> **) Inula_britanica_MeOH_15_75_NEG.d
191.0532
353.0852
Counts vs. Mass-to-Charge (m/z)

5.

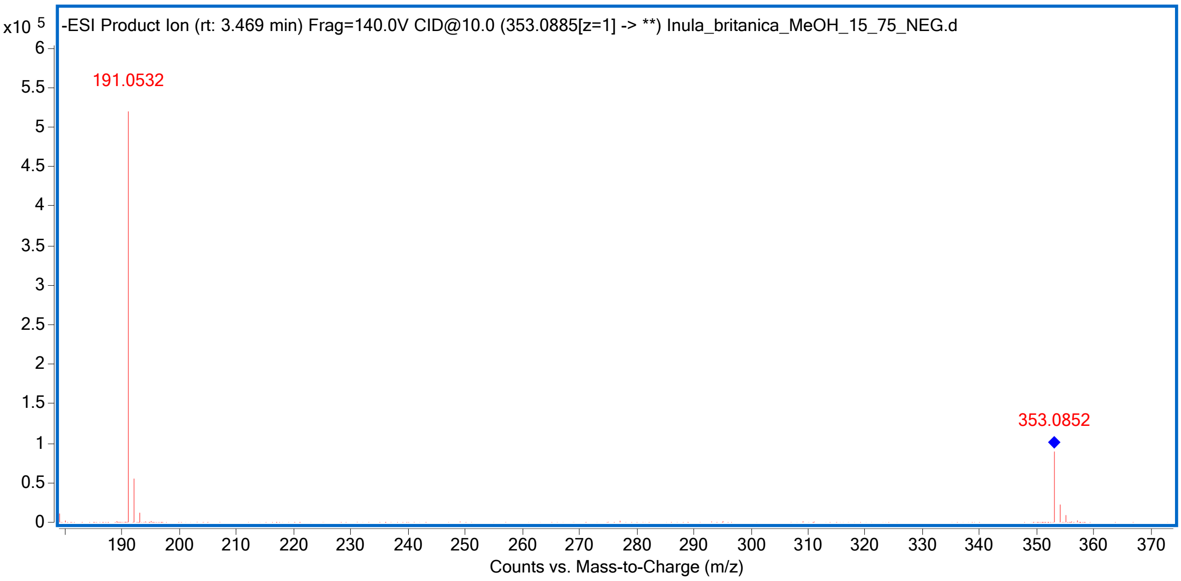
x10 5
-ESI Product Ion (rt: 3.469 min) Frag=140.0V CID@10.0 (353.0885[z=1] -> **) Inula_britanica_MeOH_15_75_NEG.d
191.0532
353.0852
Counts vs. Mass-to-Charge (m/z)

6.

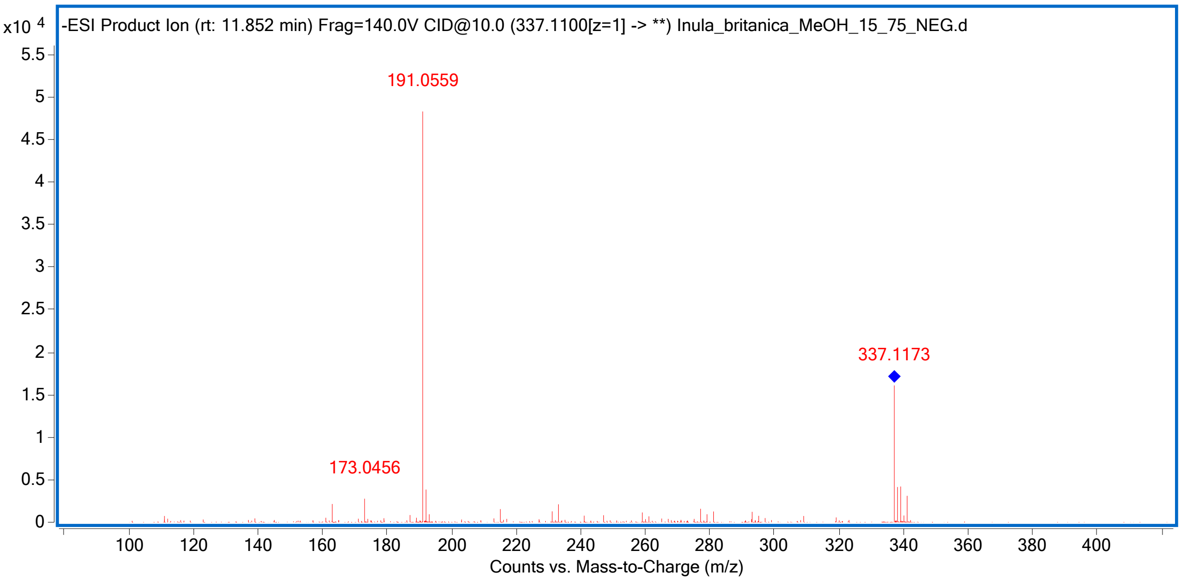
x10 4
-ESI Product Ion (rt: 11.852 min) Frag=140.0V CID@10.0 (337.1100[z=1] -> **) Inula_britanica_MeOH_15_75_NEG.d
191.0559
337.1173
173.0456
Counts vs. Mass-to-Charge (m/z)

7.

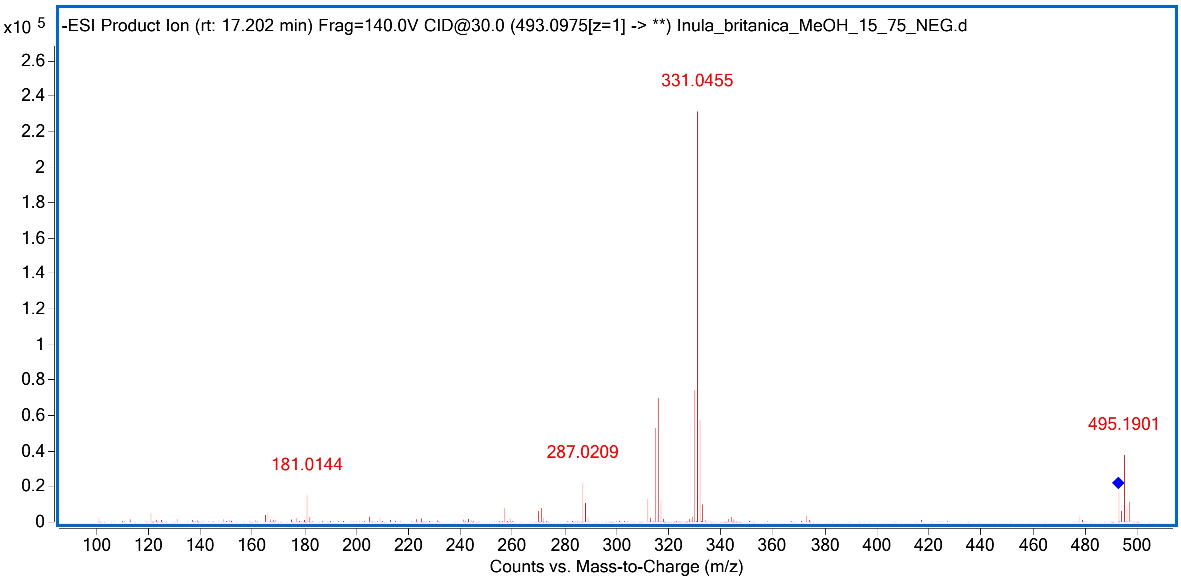
x10 5
-ESI Product Ion (rt: 17.202 min) Frag=140.0V CID@30.0 (493.0975[z=1] -> **) Inula_britanica_MeOH_15_75_NEG.d
331.0455
495.1901
287.0209
181.0144
Counts vs. Mass-to-Charge (m/z)

8.

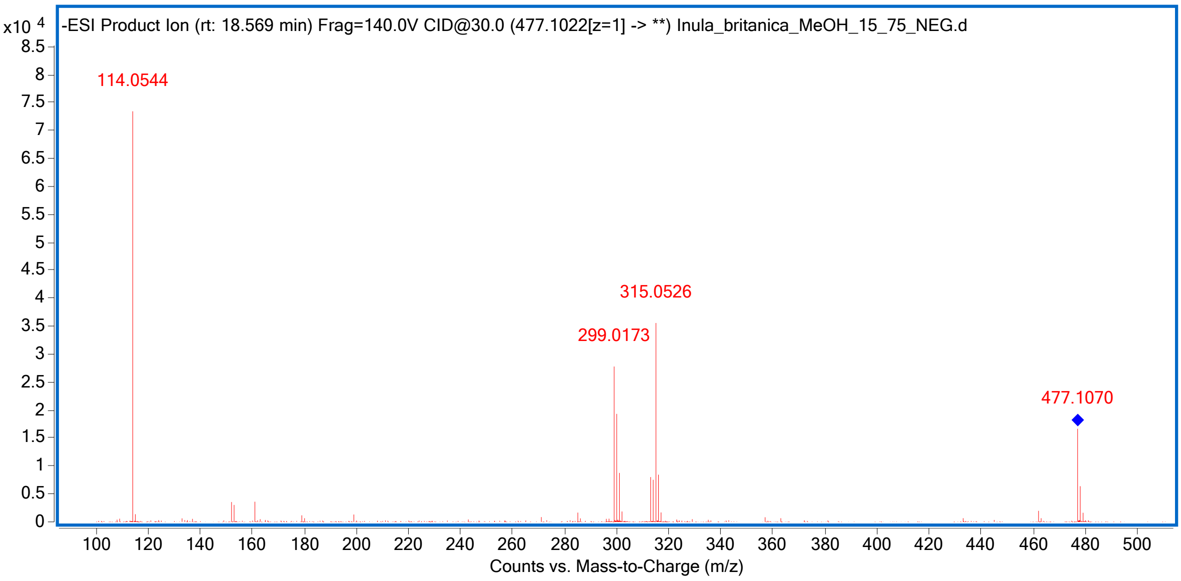
x10 4
-ESI Product Ion (rt: 18.569 min) Frag=140.0V CID@30.0 (477.1022[z=1] -> **) Inula_britanica_MeOH_15_75_NEG.d
114.0544
315.0526
299.0173
477.1070
Counts vs. Mass-to-Charge (m/z)

9.

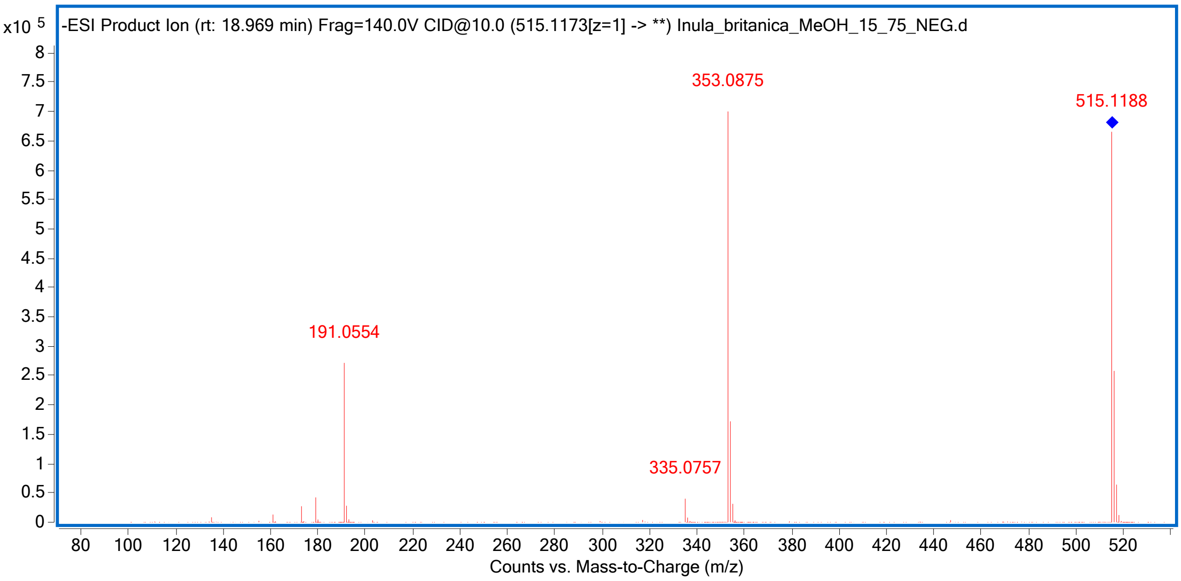
x10 5
-ESI Product Ion (rt: 18.969 min) Frag=140.0V CID@10.0 (515.1173[z=1] -> **) Inula_britanica_MeOH_15_75_NEG.d
353.0875
515.1188
191.0554
335.0757
Counts vs. Mass-to-Charge (m/z)

10.

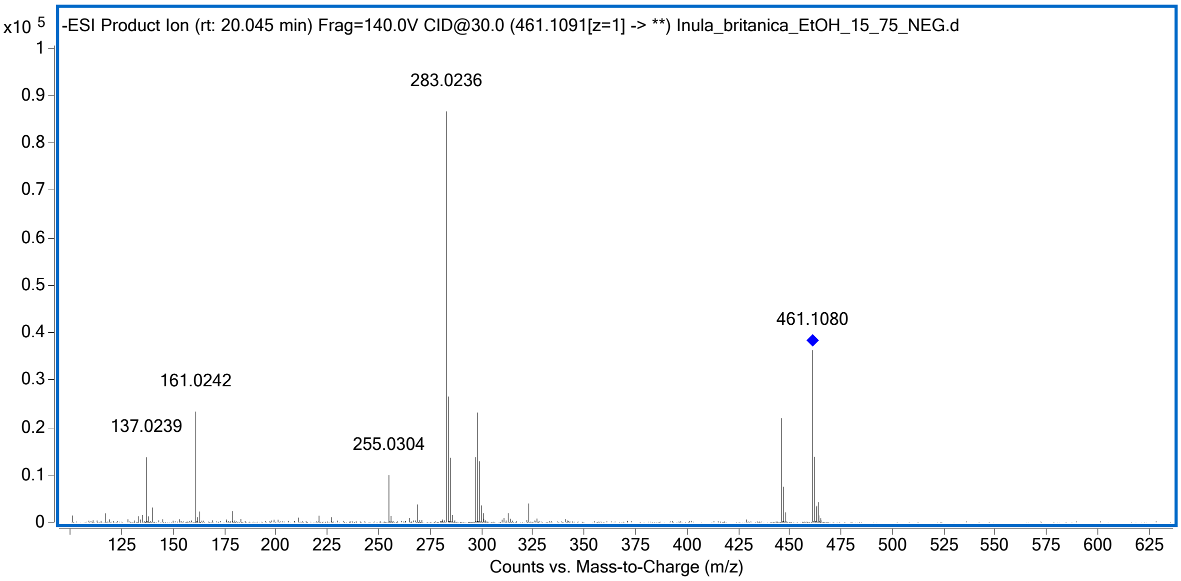
x10 5
-ESI Product Ion (rt: 20.045 min) Frag=140.0V CID@30.0 (461.1091[z=1] -> **) Inula_britanica_EtOH_15_75_NEG.d
283.0236
461.1080
161.0242
137.0239
255.0304
Counts vs. Mass-to-Charge (m/z)

11.

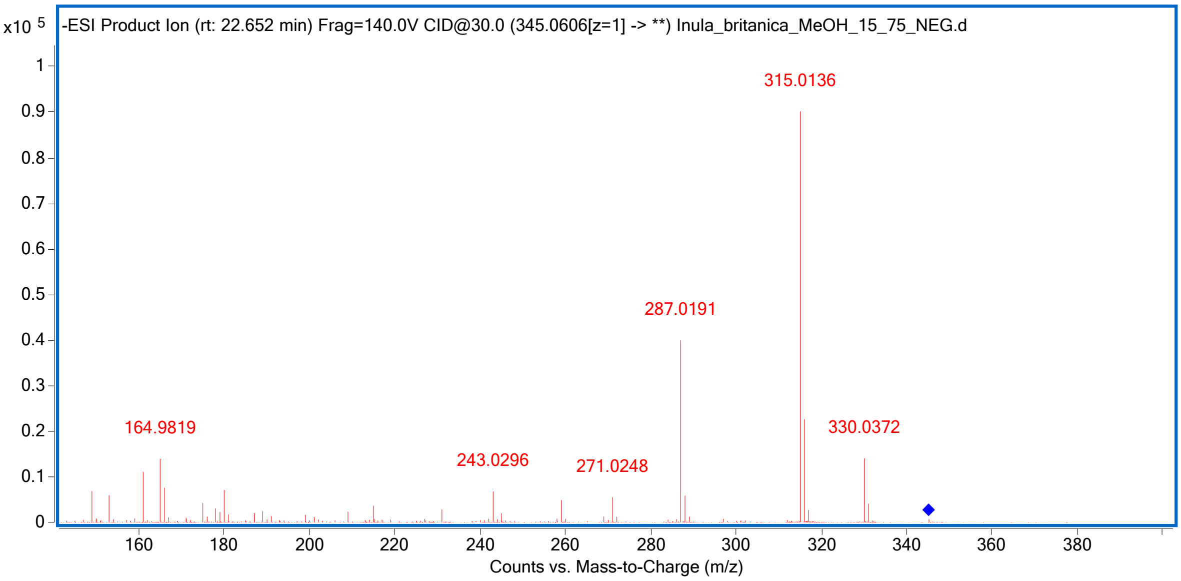
x10 5
-ESI Product Ion (rt: 22.652 min) Frag=140.0V CID@30.0 (345.0606[z=1] -> **) Inula_britanica_MeOH_15_75_NEG.d
315.0136
287.0191
164.9819
330.0372
243.0296
271.0248
Counts vs. Mass-to-Charge (m/z)

12.

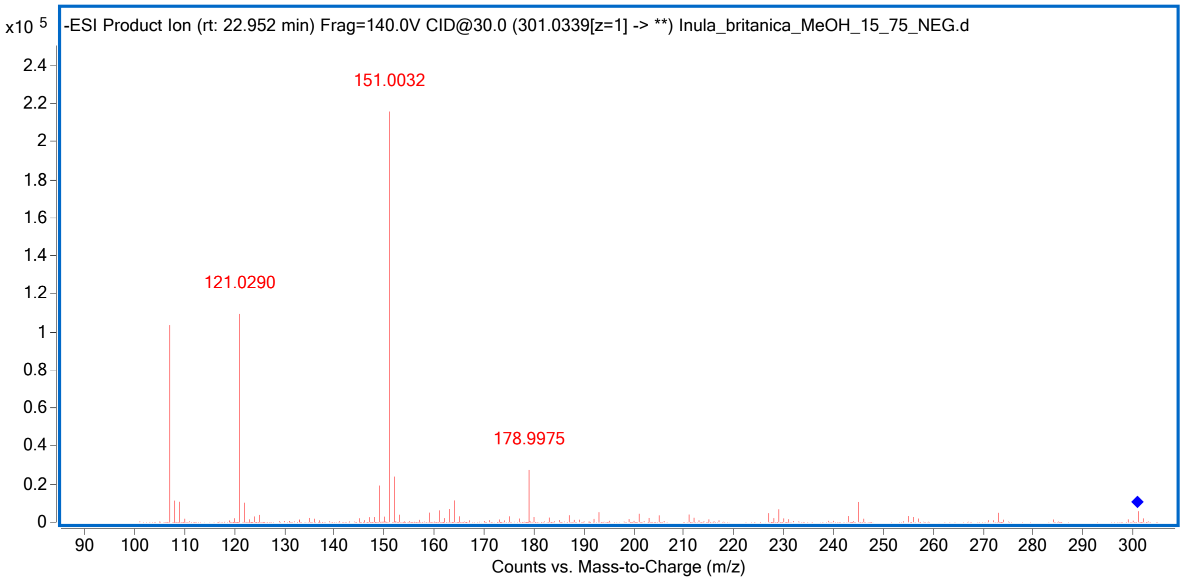
x10 5
-ESI Product Ion (rt: 22.952 min) Frag=140.0V CID@30.0 (301.0339[z=1] -> **) Inula_britanica_MeOH_15_75_NEG.d
151.0032
121.0290
178.9975
Counts vs. Mass-to-Charge (m/z)

13.

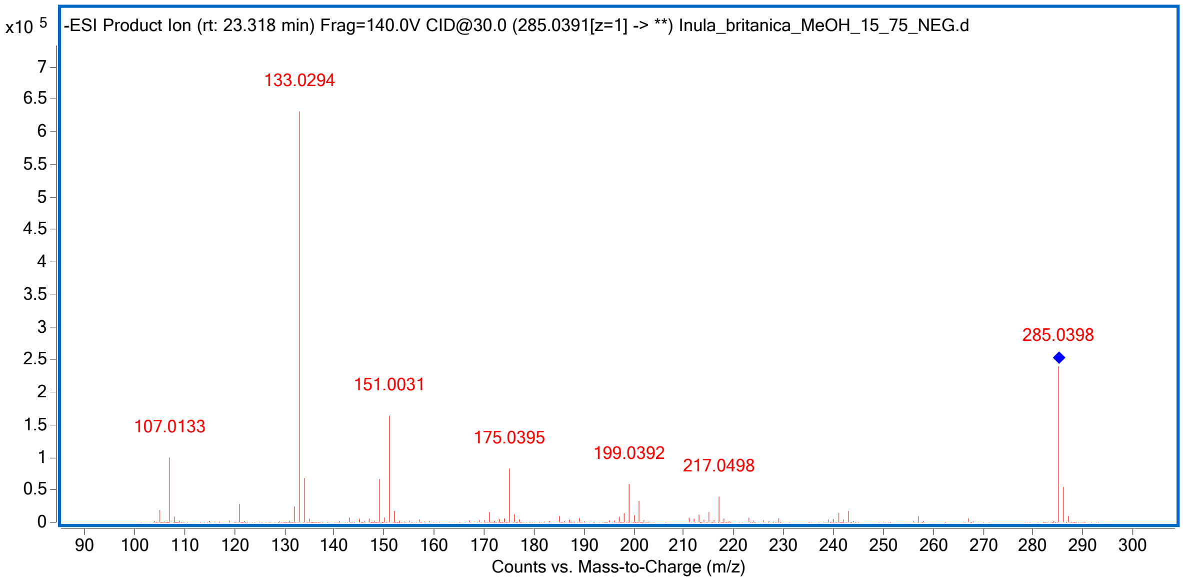
x10 5
-ESI Product Ion (rt: 23.318 min) Frag=140.0V CID@30.0 (285.0391[z=1] -> **) Inula_britanica_MeOH_15_75_NEG.d
133.0294
285.0398
151.0031
107.0133
175.0395
199.0392
217.0498
Counts vs. Mass-to-Charge (m/z)

14.

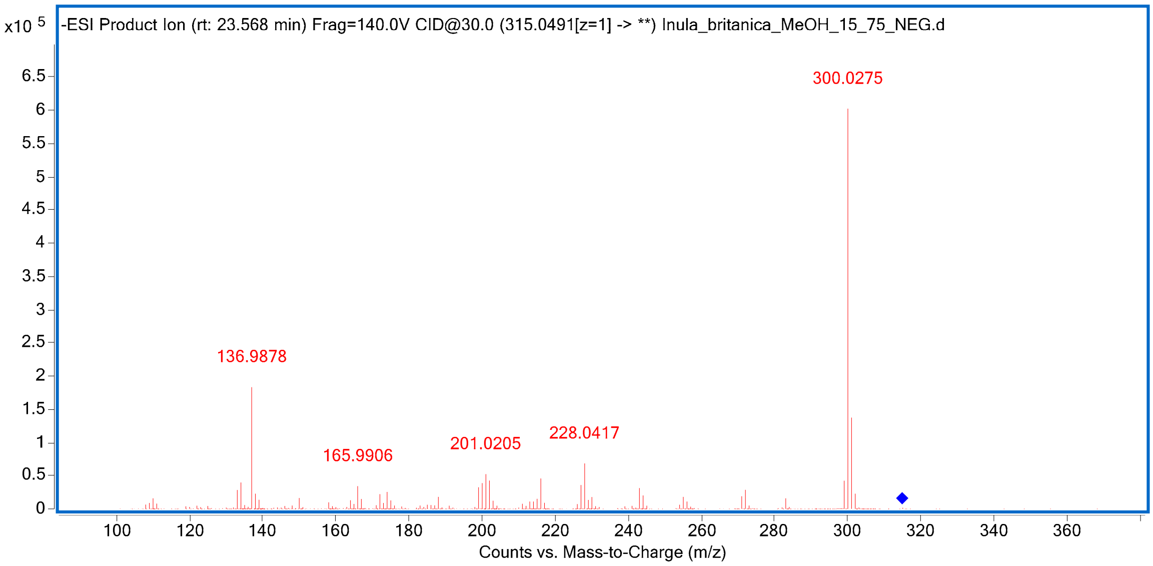
x10 5
-ESI Product Ion (rt: 23.568 min) Frag=140.0V CID@30.0 (315.0491[z=1] -> **) Inula_britanica_MeOH_15_75_NEG.d
300.0275
136.9878
165.9906
201.0205
228.0417
Counts vs. Mass-to-Charge (m/z)

15.

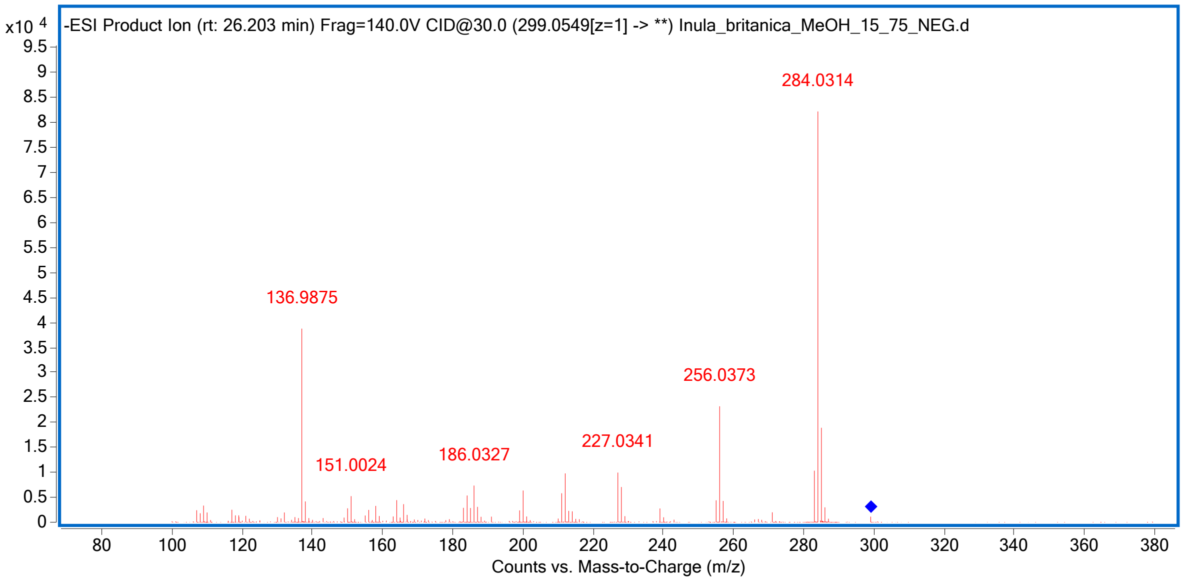

Table S1. Base peak chromatogram of *Inula britannica* L. methanolic extract by high-performance liquid chromatography-electrospray ionization-quadrupole-time of flight-mass spectrometry (HPLC/ESI-QTOF-MS) and the results of ESI-QTOF-MS analysis of *Inula britannica* L. methanolic extract.

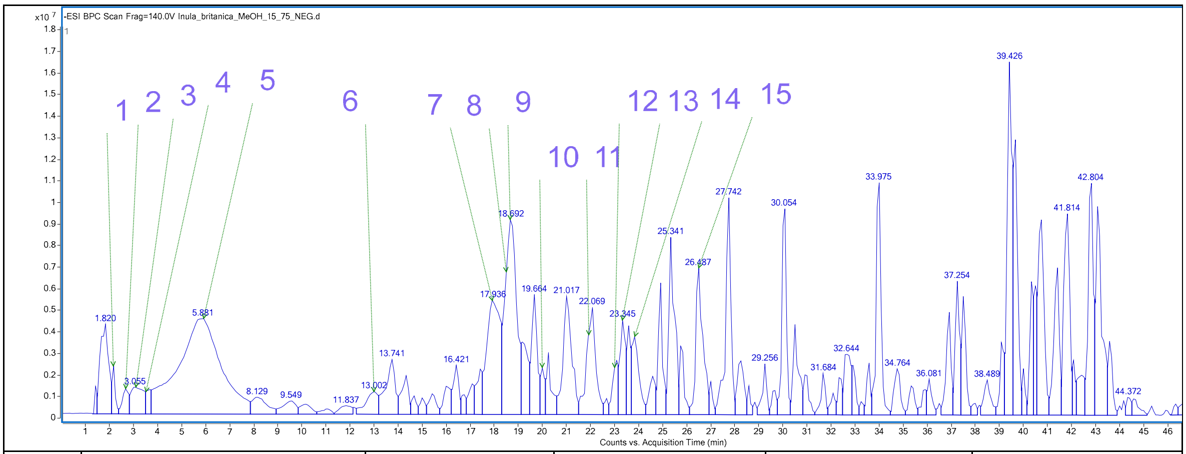

No	Tentative assignment	Retention time [min]	Formula	Molecular Ion [m/z]	MS/MS fragments [m/z]
1	Malic acid	2.063	$C_4H_6O_5$	133.0142	115.0034
2	Citric acid	2.286	$C_6H_8O_7$	191.0540	127.0412; 111.0063; 101.0592
3	Neochlorogenic acid	2.636	$C_{16}H_{18}O_9$	353.0885	191.0553 179.0342
4	Caffeic acid glucoside	3.270	$C_{15}H_{18}O_9$	341.0905	179.0304; 161.0199; 135.0408
5	Chlorogenic acid	5.986	$C_{16}H_{18}O_9$	353.0885	191.0544
6	Coumarylquinic acid isomer	12.952	$C_{16}H_{18}O_8$	337.1253	191.0554 135.0441
7	Patulitrin	17.412	$C_{22}H_{22}O_{13}$	493.0975	331.0458; 316.0210; 287.0189; 181.0137
8	Cynarine	18.162	$C_{25}H_{24}O_{11}$	515.1188	353.0875;

					191.0554
9	Nepitrin	18.212	$C_{22}H_{22}O_{12}$	477.1051	315.0704; 301.0361; 161.0239; 152.0107; 114.0550
10	Hispuduloside	20.002	$C_{22}H_{22}O_{11}$	461.1096	298.0480; 283.0232; 255.0314; 161.0255; 137.0253
11	Axillarin	21.929	$C_{17}H_{14}O_{8}$	345.0604	330.0379; 315.0142; 287.0197; 271.0244; 243.0291
12	Quercetin	23.012	$C_{15}H_{10}O_{7}$	301.0337	178.9972; 151.0030; 121.02290; 107.0133
13	Luteolin	23.328	$C_{15}H_{10}O_{6}$	285.0398	199.0395; 175.0392; 151.0034; 133.0293; 107.0134
14	Nepetin	23.869	$C_{16}H_{12}O_{7}$	315.0491	300.0267; 271.0256 243.0300; 228.0426; 216.0422; 165.9905; 136.9871
15	Kaempferol methyl ether	26.319	$C_{16}H_{12}O_{6}$	299.0549	284.0323; 256.0371; 227.0338; 151.0028

Figure S2. RP-HPLC/DAD chromatograms of *Inula britannica* L. methanolic and ethanolic extracts.

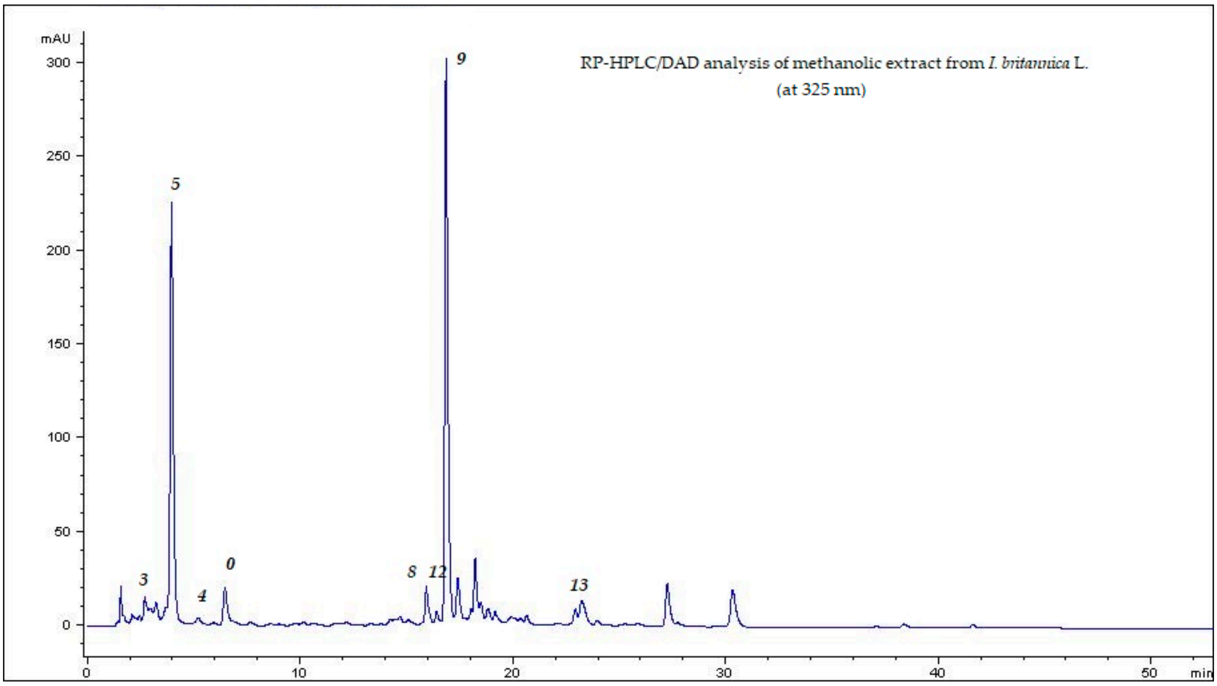
RP-HPLC/DAD analysis of methanolic extract from I. britannica L.
(at 325 nm)
mAU
300
250
200
150
100
50
0
0
10
20
30
40
50
min
3
5
4
0
8
12
9
13

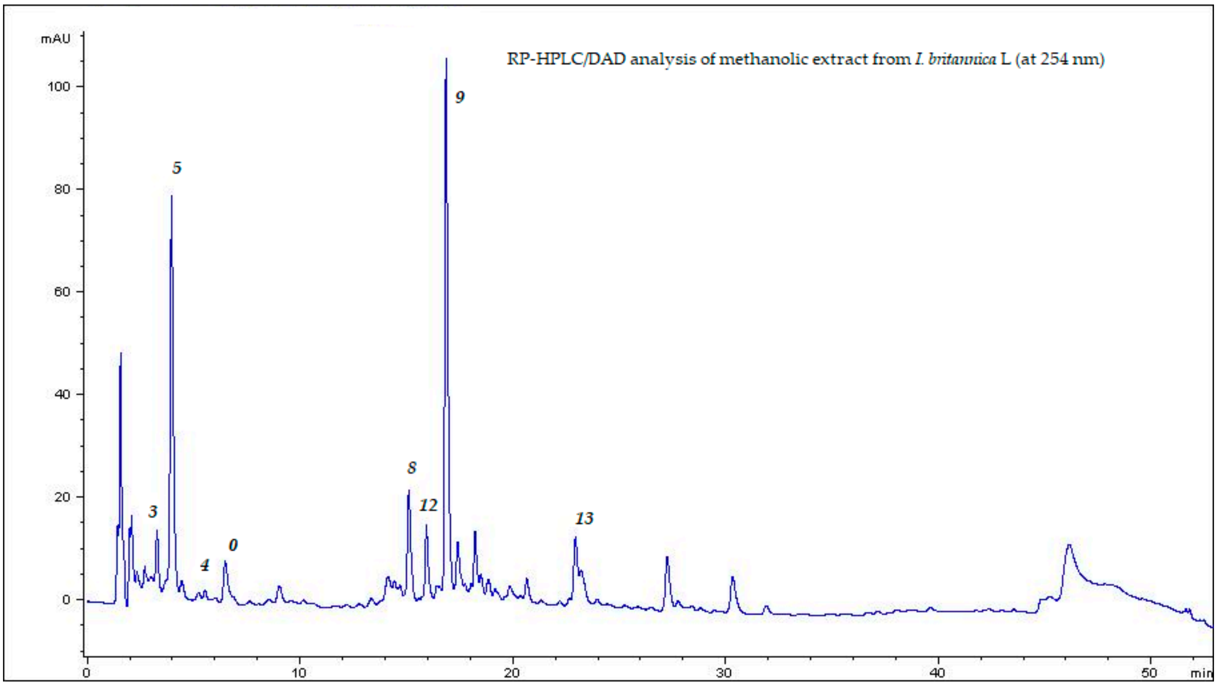
RP-HPLC/DAD analysis of methanolic extract from I. britannica L (at 254 nm)
mAU
100
80
60
40
20
0
0
10
20
30
40
50
min
3
5
4
0
8
12
9
13

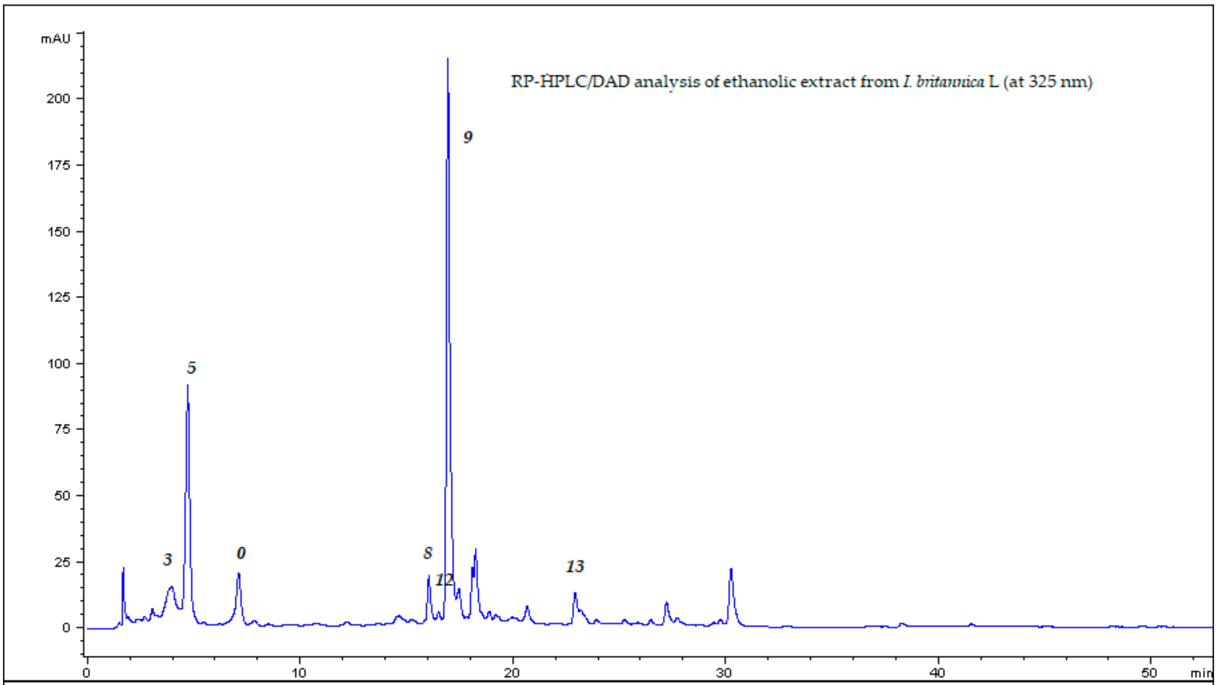
RP-HPLC/DAD analysis of ethanolic extract from *I. britannica* L (at 325 nm)
mAU
200
175
150
125
100
75
50
25
0
0
10
20
30
40
50
min
5
3
0
8
12
9
13

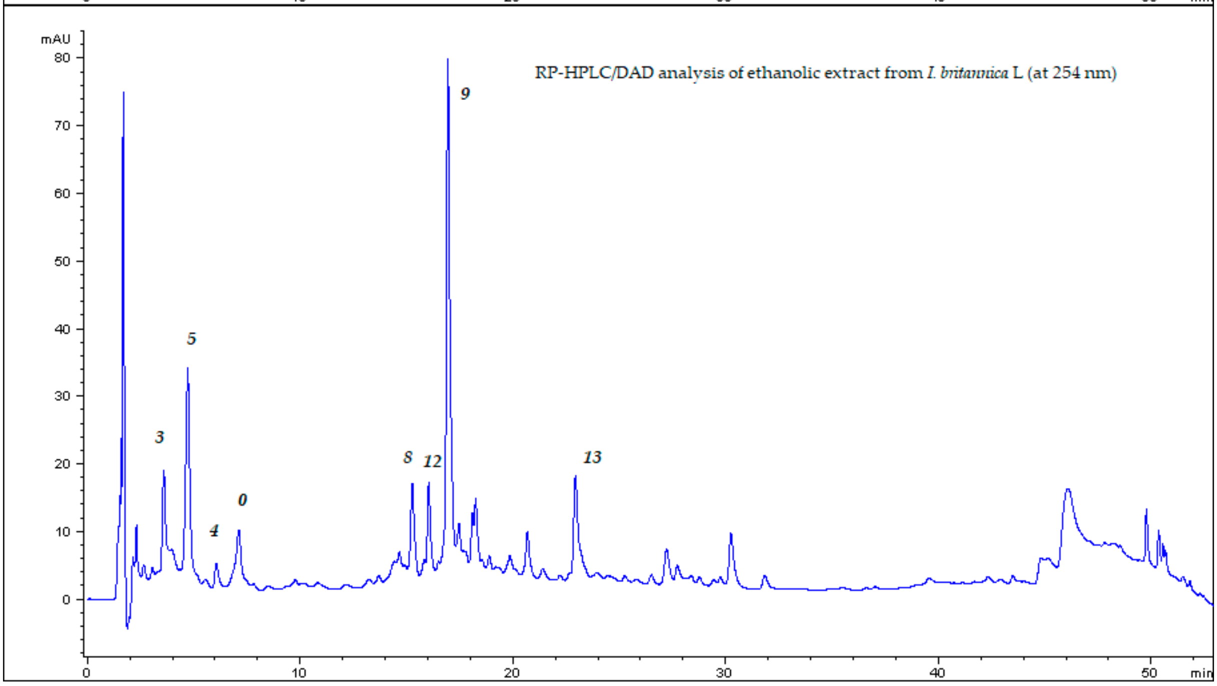
RP-HPLC/DAD analysis of ethanolic extract from *I. britannica* L (at 254 nm)
mAU
80
70
60
50
40
30
20
10
0
0
10
20
30
40
50
min
5
3
4
0
8
12
9
13
